# Supplementary figures and images for: Immune subtyping of lymph node metastasis-negative colorectal cancer reveals biomarkers for prognosis and immunotherapy response
Source: PLoS One. 2025 Sep 24;20(9):e0332915. doi: 10.1371/journal.pone.0332915 (PMC12459767; doi:10.1371/journal.pone.0332915)

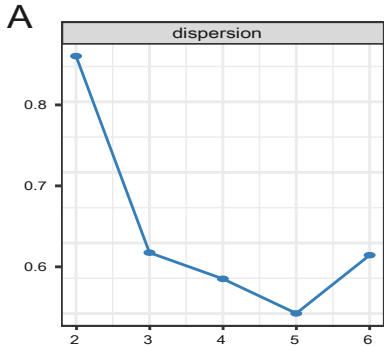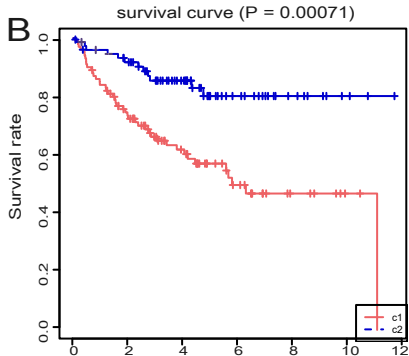

Supplement: S1 Fig — (A) Use immune-related genes to perform NMF clustering on the GSE103479. Cophenetic correlation coefficient for k = 2–6 is shown. (B) Overall survival (OS) analysis of the two subclasses (C1 and C2) in GSE103479, with statistical significance assessed by log-rank test. (PDF) [file pone.0332915.s011.pdf]

*liprin a4*(134kd)

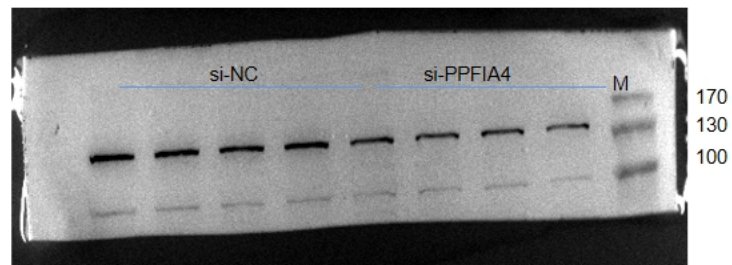

*GAPDH* (36kd)

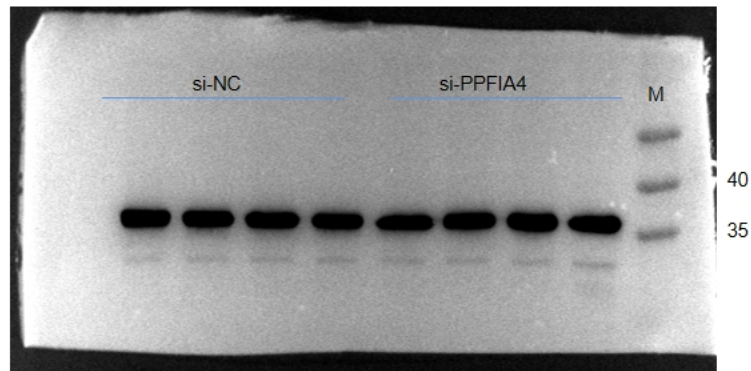

SW480

*liprin a4*(134kd)

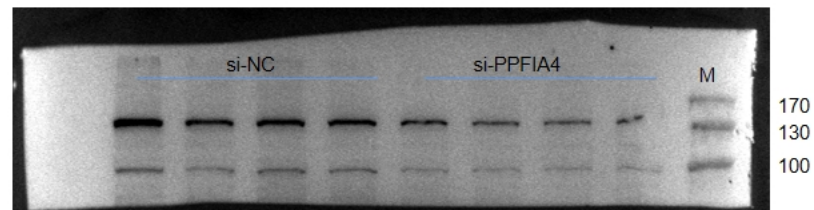

*GAPDH* (36kd)

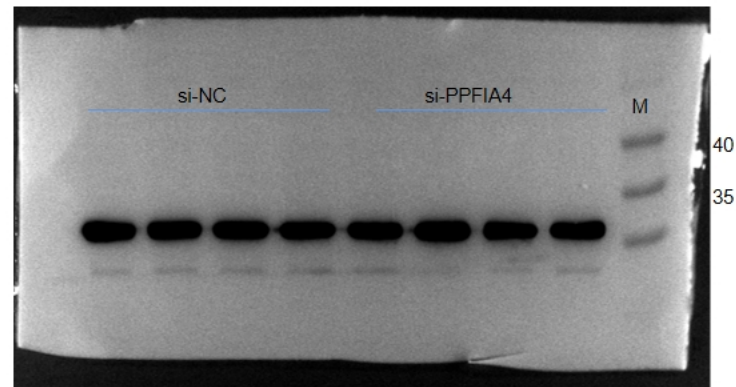

HCT-116

Supplement: S1 File — Original, uncropped Western blot images corresponding to the results shown in Figure 6B (liprin a4 and GAPDH in SW480 and HCT-116 cells). (PDF) [file pone.0332915.s012.pdf]
