# Supplementary material for: Immune subtyping of lymph node metastasis-negative colorectal cancer reveals biomarkers for prognosis and immunotherapy response
Source: PLoS One. 2025 Sep 24;20(9):e0332915. doi: 10.1371/journal.pone.0332915 (PMC12459767; doi:10.1371/journal.pone.0332915)
Supplement: Attachment — Submitted filename: Response to Reviewers.docx [file pone.0332915.s015.docx]

**Journal Requirements:**

1.Please ensure that your manuscript meets PLOS ONE's style requirements, including those for file naming. The PLOS ONE style templates can be found at https://journals.plos.org/plosone/s/file?id=wjVg/PLOSOne_formatting_sample_main_body.pdf and

https://journals.plos.org/plosone/s/file?id=ba62/PLOSOne_formatting_sample_title_authors_affiliations.pdf

Response: Done.

2. Please note that funding information should not appear in any section or other areas of your manuscript. We will only publish funding information present in the Funding Statement section of the online submission form. Please remove any funding-related text from the manuscript.

Response: Done.

3. We note that the grant information you provided in the ‘Funding Information’ and ‘Financial Disclosure’ sections do not match.

When you resubmit, please ensure that you provide the correct grant numbers for the awards you received for your study in the ‘Funding Information’ section.

Response: Thank you for pointing out the discrepancy. We have reviewed and corrected the grant information to ensure consistency between the ‘Funding Information’ and ‘Financial Disclosure’ sections. The correct grant number(s) and funding agency details will be clearly provided in the revised submission.

4. When completing the data availability statement of the submission form, you indicated that you will make your data available on acceptance. We strongly recommend all authors decide on a data sharing plan before acceptance, as the process can be lengthy and hold up publication timelines. Please note that, though access restrictions are acceptable now, your entire data will need to be made freely accessible if your manuscript is accepted for publication. This policy applies to all data except where public deposition would breach compliance with the protocol approved by your research ethics board. If you are unable to adhere to our open data policy, please kindly revise your statement to explain your reasoning and we will seek the editor's input on an exemption. Please be assured that, once you have provided your new statement, the assessment of your exemption will not hold up the peer review process.

Response: Thank you for your reminder. We fully understand and respect PLOS ONE’s open data policy. We have prepared all underlying data and are currently organizing it for public deposition. Upon acceptance, we plan to make the complete dataset freely accessible through an established public repository such as [e.g., Figshare, Dryad, GEO, or Zenodo]. We are committed to ensuring that all data necessary to replicate and validate our findings will be openly available prior to publication.

Should you require any further clarification or recommendations regarding the most suitable repository for our data type, we would be happy to comply.

5. Please include captions for your Supporting Information files at the end of your manuscript, and update any in-text citations to match accordingly. Please see our Supporting Information guidelines for more information: [http://journals.plos.org/plosone/s/supporting-information](http://track.editorialmanager.com/CL0/http:%2F%2Fjournals.plos.org%2Fplosone%2Fs%2Fsupporting-information/1/010f01969e3ebbe0-78bad238-6292-42e5-b879-fe5acd077e19-000000/2uSw4M0bjcIlyTcHd1F4RzE3tEhMKSAew0nkxQ3ljyA=209).

Response: Done.

6. PLOS ONE now requires that authors provide the original uncropped and unadjusted images underlying all blot or gel results reported in a submission’s figures or Supporting Information files. This policy and the journal’s other requirements for blot/gel reporting and figure preparation are described in detail at <https://journals.plos.org/plosone/s/figures#loc-blot-and-gel-reporting-requirements> and <https://journals.plos.org/plosone/s/figures#loc-preparing-figures-from-image-files>. When you submit your revised manuscript, please ensure that your figures adhere fully to these guidelines and provide the original underlying images for all blot or gel data reported in your submission. See the following link for instructions on providing the original image data: <https://journals.plos.org/plosone/s/figures#loc-original-images-for-blots-and-gels>.

In your cover letter, please note whether your blot/gel image data are in Supporting Information or posted at a public data repository, provide the repository URL if relevant, and provide specific details as to which raw blot/gel images, if any, are not available. Email us at plosone@plos.org if you have any questions.

Response: We have prepared the original, uncropped, and unadjusted images underlying all blot and gel results reported in the manuscript. These files have been compiled into a PDF document and are provided as Supporting Information in accordance with the journal’s guidelines. Please let us know if further formatting or additional information is required.

**Reviewers' comments:**
1. Is the manuscript technically sound, and do the data support the conclusions?

The manuscript must describe a technically sound piece of scientific research with data that supports the conclusions. Experiments must have been conducted rigorously, with appropriate controls, replication, and sample sizes. The conclusions must be drawn appropriately based on the data presented.

Reviewer #1: Yes

Reviewer #2: Yes

2. Has the statistical analysis been performed appropriately and rigorously?

Reviewer #1: I Don't Know

Reviewer #2: Yes

3. Have the authors made all data underlying the findings in their manuscript fully available?

The [PLOS Data policy](http://www.plosone.org/static/policies.action#sharing) requires authors to make all data underlying the findings described in their manuscript fully available without restriction, with rare exception (please refer to the Data Availability Statement in the manuscript PDF file). The data should be provided as part of the manuscript or its supporting information, or deposited to a public repository. For example, in addition to summary statistics, the data points behind means, medians and variance measures should be available. If there are restrictions on publicly sharing data—e.g. participant privacy or use of data from a third party—those must be specified.

Reviewer #1: Yes

Reviewer #2: Yes

4. Is the manuscript presented in an intelligible fashion and written in standard English?

PLOS ONE does not copyedit accepted manuscripts, so the language in submitted articles must be clear, correct, and unambiguous. Any typographical or grammatical errors should be corrected at revision, so please note any specific errors here.

Reviewer #1: Yes

Reviewer #2: Yes

5. Review Comments to the Author

Please use the space provided to explain your answers to the questions above. You may also include additional comments for the author, including concerns about dual publication, research ethics, or publication ethics. (Please upload your review as an attachment if it exceeds 20,000 characters)

Reviewer #1: This manuscript describes an interesting and relevant study on immune subtyping of lymph node metastasis-negative colorectal cancer, which holds great promise for providing biomarkers to predict prognosis and response to immunotherapy. The experimental design seems appropriate, and the conclusions drawn are generally supported by the data.However, I recommend statistical analysis experts who can better comment on this aspect, given that I am not a statistics professional and cannot fully evaluate the rigor of this component.

Recommendations:
• The methodology should discuss quite well the choice of specific machine learning algorithms.

Response: We appreciate the reviewer’s insightful comment. In the revised Materials and Methods section, we have clarified the rationale for selecting each machine learning algorithm within the Materials and Methods section. Specifically, we used NMF for its effectiveness in identifying biologically meaningful, non-overlapping expression patterns for cancer subtyping. PCA was then employed to construct the ICI score by extracting principal components that summarize key feature dimensions into interpretable scores. These revisions now better justify our methodological choices.

• Compare the results with those of existing classifications of colorectal cancer

Response: Thank you for the helpful suggestion. A brief comparison with existing classification systems, such as CMS, has been added at the end of the first paragraph of the Discussion section. Our findings indicate that the proposed immune-based subtyping offers more precise stratification, particularly for LNM-negative CRC patients, and serves as a complementary approach to the broader transcriptomic-based classifications.

• Strengthen the discussion of clinical applications, the sense of the clinical contributions of PPFIA4 and ICI scores should be amplified.

Response: We appreciate the reviewer’s suggestion. In the revised Discussion section, we have expanded the discussion on the clinical implications of PPFIA4 and the ICI score. Specifically, PPFIA4 is highlighted as a potential diagnostic and therapeutic target, given its subtype-specific expression profile and involvement in CRC progression. Moreover, the ICI score is emphasized as a practical immune-related metric, capable of identifying LNM-negative patients who may derive benefit from immunotherapy. This metric may complement current biomarkers and guide personalized treatment decisions. These revisions underscore the translational potential of our findings.

• Improve the quality of the figures, as they appear blurry.

Response: We thank the reviewer for pointing out the issue regarding figure clarity. In response, we have replaced the affected figures with high-resolution versions to ensure clarity and compliance with publication standards. We believe these improvements substantially enhance the overall visual presentation of our data.

Reviewer #2: Dear authors,

Thank you for submitting this interesting and timely research. Please find below my comments aimed at improving clarity and strengthening the manuscript:

Clarification of Subtype Characteristics:
The subtypes C1 and C2 are defined, but their prognostic and immunological implications remain somewhat unclear. Both are described as having "high immune infiltration" or "favorable prognosis," which can be confusing. Please consider clearly and consistently presenting the defining features—both prognostic and immunological—of each subtype early in the manuscript and maintain this consistency throughout.

Contextualization with Existing Classifications:
It would be helpful to expand on how the C1/C2 subtypes align with or differ from existing colorectal cancer immune classifications, such as the CMS (Consensus Molecular Subtypes). Additionally, consider discussing the broader relevance to immunotherapy beyond PD-1/PD-L1, and how the ICI scores proposed in your study might guide treatment decisions in clinical practice.

Limitations Section:
Please include a dedicated limitations section and elaborate on the following points:
The lack of in vivo validation for PPFIA4.

Response: We thank the reviewer for highlighting this important limitation. As our current study primarily employed in vitro assays, we now explicitly acknowledge in the Discussion that in vivo validation of PPFIA4’s functional role and therapeutic relevance remains essential. We have also outlined the need for future studies using animal models or patient-derived samples to confirm our findings. This acknowledgment clarifies the scope of the current study and sets a clear direction for future research.

The potential for dataset bias (GEO vs. TCGA).

Response: We thank the reviewer for pointing out the important issue of potential dataset bias between TCGA and GEO. To minimize such bias, we performed the following measures:

1)Independent Subtype Identification and Validation: The immune subtypes (C1 and C2) were separately identified in the TCGA dataset and independently validated in two GEO datasets (GSE39582 and GSE103479). The consistent results across datasets (confirmed by NMF clustering, t-SNE analysis, and survival differences) demonstrate the robustness and reproducibility of the subtype classification.

2)Standard Preprocessing: All datasets underwent standardized preprocessing procedures, including normalization and log2 transformation. Low-variance genes were removed prior to clustering to reduce noise.

3)Cross-dataset Consistency: In both TCGA and GEO cohorts, the subtypes showed similar immune characteristics, prognostic trends, and immune checkpoint profiles, which reduces the likelihood of dataset-specific artifacts.

4)Additional Clarification: In the revised manuscript (Results section), we have added a brief explanation on how dataset bias was addressed, and emphasized the use of independent validation cohorts to strengthen our conclusions.

These steps enhance confidence in the reliability and transferability of our findings.

The generalizability of your findings across diverse CRC patient populations.
Response: We thank the reviewer for raising this important point.. Our study specifically focused on LNM-negative CRC patients to minimize confounding factors associated with advanced disease stages. We acknowledge that the immune microenvironment may differ significantly in LNM-positive patients, potentially affecting the applicability of our identified immune subtypes. We have addressed this limitation in the revised manuscript and emphasized the need for future studies to validate our findings in broader CRC populations, including those with lymph node involvement.

Language and Formatting:
The manuscript would benefit from a careful grammar and spell check, as well as the correction of typographical errors throughout.

Response: We thank the reviewer for noting the need for language and formatting improvements. In response, we have thoroughly reviewed the manuscript to correct grammatical errors, spelling mistakes, and typographical issues. Additionally, we have ensured consistency in formatting throughout the document. We believe these revisions have enhanced the clarity and readability of the manuscript.

6. PLOS authors have the option to publish the peer review history of their article ([what does this mean?](https://journals.plos.org/plosone/s/editorial-and-peer-review-process#loc-peer-review-history)). If published, this will include your full peer review and any attached files.


If you choose “no”, your identity will remain anonymous but your review may still be made public.


**Do you want your identity to be public for this peer review?** For information about this choice, including consent withdrawal, please see our [Privacy Policy](https://www.plos.org/privacy-policy).

Reviewer #1: No

Reviewer #2: No
