# Supplementary material for: Immune subtyping of lymph node metastasis-negative colorectal cancer reveals biomarkers for prognosis and immunotherapy response
Source: PLoS One. 2025 Sep 24;20(9):e0332915. doi: 10.1371/journal.pone.0332915 (PMC12459767; doi:10.1371/journal.pone.0332915)
Supplement: Attachment — Submitted filename: PONE-D-25-00542_R1_rev.docx [file pone.0332915.s014.docx]

**PLOS ONE**

**Immune Subtyping of Lymph Node Metastasis-Negative Colorectal Cancer Reveals Biomarkers for Prognosis and Immunotherapy Response** --Manuscript Draft--

**Manuscript Number:**

**Article Type:**

**Full Title:**

**Short Title:**

**Corresponding Author:**

**Keywords:**

**Abstract:**

PONE-D-25-00542R1

Research Article

Immune Subtyping of Lymph Node Metastasis-Negative Colorectal Cancer Reveals Biomarkers for Prognosis and Immunotherapy Response

Immune Profiling of Lymph Node-Negative Colorectal Cancer

Wenliang Yuan Jiaxing University Jiaxing, CHINA

CRC; immunotherapy; LNM-negative; PPFIA4

Background

Lymph node metastasis (LNM) is a key prognostic factor in colorectal cancer (CRC), and early lymph node status assessment is crucial for prognosis and immunotherapy decisions. However, the immune characteristics of LNM-negative CRC remain poorly understood.

Methods

Using machine learning algorithms, we identified and analyzed two immune subtypes (C1 and C2) in 244 LNM-negative CRC samples, with validation in 458 additional samples, and evaluated their immune characteristics and functional pathways.

Results

Subtype C1 exhibited high immune scores and responsiveness to immune checkpoint blockade, while subtype C2 showed a favorable prognosis and increased immune cell infiltration, indicating it represents an earlier CRC stage. Experimental validation revealed that PPFIA4 knockdown in C1 significantly suppressed CRC cell proliferation and migration.

Conclusions

**Order of Authors:**

**Response to Reviewers:**

These findings provide insights into personalized immunotherapy strategies for early-stage CRC patients and have potential clinical application value.

Wenliang Yuan

Li Liu

Journal Requirements:

1.Please ensure that your manuscript meets PLOS ONE's style requirements, including those for file naming. The PLOS ONE style templates can be found at https://journals.plos.org/plosone/s/file?id=wjVg/PLOSOne_formatting_sample_main_bo dy.pdf and https://journals.plos.org/plosone/s/file?id=ba62/PLOSOne_formatting_sample_title_aut hors_affiliations.pdf

Response: Done.

2. Please note that funding information should not appear in any section or other areas of your manuscript. We will only publish funding information present in the Funding Statement section of the online submission form. Please remove any funding-related text from the manuscript.

Response: Done.

3. We note that the grant information you provided in the ‘Funding Information’ and ‘Financial Disclosure’ sections do not match.

When you resubmit, please ensure that you provide the correct grant numbers for the

*Powered by Editorial Manager® and ProduXion Manager® from Aries Systems Corporation*

awards you received for your study in the ‘Funding Information’ section. Response: Thank you for pointing out the discrepancy. We have reviewed and corrected the grant information to ensure consistency between the ‘Funding Information’ and ‘Financial Disclosure’ sections. The correct grant number(s) and funding agency details will be clearly provided in the revised submission.

4. When completing the data availability statement of the submission form, you indicated that you will make your data available on acceptance. We strongly recommend all authors decide on a data sharing plan before acceptance, as the process can be lengthy and hold up publication timelines. Please note that, though access restrictions are acceptable now, your entire data will need to be made freely accessible if your manuscript is accepted for publication. This policy applies to all data except where public deposition would breach compliance with the protocol approved by your research ethics board. If you are unable to adhere to our open data policy, please kindly revise your statement to explain your reasoning and we will seek the editor's input on an exemption. Please be assured that, once you have provided your new statement, the assessment of your exemption will not hold up the peer review process.

Response: Thank you for your reminder. We fully understand and respect PLOS ONE’s open data policy. We have prepared all underlying data and are currently organizing it for public deposition. Upon acceptance, we plan to make the complete dataset freely accessible through an established public repository such as [e.g., Figshare, Dryad, GEO, or Zenodo]. We are committed to ensuring that all data necessary to replicate and validate our findings will be openly available prior to publication.

Should you require any further clarification or recommendations regarding the most suitable repository for our data type, we would be happy to comply.

5. Please include captions for your Supporting Information files at the end of your manuscript, and update any in-text citations to match accordingly. Please see our Supporting Information guidelines for more information: http://journals.plos.org/plosone/s/supporting-information.

Response: Done.

6. PLOS ONE now requires that authors provide the original uncropped and unadjusted images underlying all blot or gel results reported in a submission’s figures or Supporting Information files. This policy and the journal’s other requirements for blot/gel reporting and figure preparation are described in detail at https://journals.plos.org/plosone/s/figures#loc-blot-and-gel-reporting-requirements and https://journals.plos.org/plosone/s/figures#loc-preparing-figures-from-image-files. When you submit your revised manuscript, please ensure that your figures adhere fully to these guidelines and provide the original underlying images for all blot or gel data reported in your submission. See the following link for instructions on providing the original image data: https://journals.plos.org/plosone/s/figures#loc-original-images-for-blots-and-gels.

In your cover letter, please note whether your blot/gel image data are in Supporting Information or posted at a public data repository, provide the repository URL if relevant, and provide specific details as to which raw blot/gel images, if any, are not available. Email us at plosone@plos.org if you have any questions.

Response: We have prepared the original, uncropped, and unadjusted images underlying all blot and gel results reported in the manuscript. These files have been compiled into a PDF document and are provided as Supporting Information in accordance with the journal’s guidelines. Please let us know if further formatting or additional information is required.

Reviewers' comments:

1. Is the manuscript technically sound, and do the data support the conclusions?

The manuscript must describe a technically sound piece of scientific research with data that supports the conclusions. Experiments must have been conducted rigorously, with appropriate controls, replication, and sample sizes. The conclusions must be drawn appropriately based on the data presented.

Reviewer #1: Yes Reviewer #2: Yes

________________________________________

2. Has the statistical analysis been performed appropriately and rigorously? Reviewer #1: I Don't Know

Reviewer #2: Yes

*Powered by Editorial Manager® and ProduXion Manager® from Aries Systems Corporation*

________________________________________

3. Have the authors made all data underlying the findings in their manuscript fully available?

The PLOS Data policy requires authors to make all data underlying the findings described in their manuscript fully available without restriction, with rare exception (please refer to the Data Availability Statement in the manuscript PDF file). The data should be provided as part of the manuscript or its supporting information, or deposited to a public repository. For example, in addition to summary statistics, the data points behind means, medians and variance measures should be available. If there are restrictions on publicly sharing data—e.g. participant privacy or use of data from a third party—those must be specified.

Reviewer #1: Yes Reviewer #2: Yes

________________________________________

4. Is the manuscript presented in an intelligible fashion and written in standard English?

PLOS ONE does not copyedit accepted manuscripts, so the language in submitted articles must be clear, correct, and unambiguous. Any typographical or grammatical errors should be corrected at revision, so please note any specific errors here. Reviewer #1: Yes

Reviewer #2: Yes ________________________________________ 5. Review Comments to the Author

Please use the space provided to explain your answers to the questions above. You may also include additional comments for the author, including concerns about dual publication, research ethics, or publication ethics. (Please upload your review as an attachment if it exceeds 20,000 characters)

Reviewer #1: This manuscript describes an interesting and relevant study on immune subtyping of lymph node metastasis-negative colorectal cancer, which holds great promise for providing biomarkers to predict prognosis and response to immunotherapy. The experimental design seems appropriate, and the conclusions drawn are generally supported by the data.However, I recommend statistical analysis experts who can better comment on this aspect, given that I am not a statistics professional and cannot fully evaluate the rigor of this component.

Recommendations:

• The methodology should discuss quite well the choice of specific machine learning algorithms.

Response: We appreciate the reviewer’s insightful comment. In the revised Materials and Methods section, we have clarified the rationale for selecting each machine learning algorithm within the Materials and Methods section. Specifically, we used NMF for its effectiveness in identifying biologically meaningful, non-overlapping expression patterns for cancer subtyping. PCA was then employed to construct the ICI score by extracting principal components that summarize key feature dimensions into interpretable scores. These revisions now better justify our methodological choices.

• Compare the results with those of existing classifications of colorectal cancer Response: Thank you for the helpful suggestion. A brief comparison with existing classification systems, such as CMS, has been added at the end of the first paragraph of the Discussion section. Our findings indicate that the proposed immune-based subtyping offers more precise stratification, particularly for LNM-negative CRC patients, and serves as a complementary approach to the broader transcriptomic-based classifications.

• Strengthen the discussion of clinical applications, the sense of the clinical contributions of PPFIA4 and ICI scores should be amplified.

Response: We appreciate the reviewer’s suggestion. In the revised Discussion section, we have expanded the discussion on the clinical implications of PPFIA4 and the ICI score. Specifically, PPFIA4 is highlighted as a potential diagnostic and therapeutic target, given its subtype-specific expression profile and involvement in CRC progression. Moreover, the ICI score is emphasized as a practical immune-related

*Powered by Editorial Manager® and ProduXion Manager® from Aries Systems Corporation*

metric, capable of identifying LNM-negative patients who may derive benefit from immunotherapy. This metric may complement current biomarkers and guide personalized treatment decisions. These revisions underscore the translational potential of our findings.

• Improve the quality of the figures, as they appear blurry.

Response: We thank the reviewer for pointing out the issue regarding figure clarity. In response, we have replaced the affected figures with high-resolution versions to ensure clarity and compliance with publication standards. We believe these improvements substantially enhance the overall visual presentation of our data. Reviewer #2: Dear authors,

Thank you for submitting this interesting and timely research. Please find below my comments aimed at improving clarity and strengthening the manuscript:

Clarification of Subtype Characteristics:

The subtypes C1 and C2 are defined, but their prognostic and immunological implications remain somewhat unclear. Both are described as having "high immune infiltration" or "favorable prognosis," which can be confusing. Please consider clearly and consistently presenting the defining features—both prognostic and

immunological—of each subtype early in the manuscript and maintain this consistency throughout.

Contextualization with Existing Classifications:

It would be helpful to expand on how the C1/C2 subtypes align with or differ from existing colorectal cancer immune classifications, such as the CMS (Consensus Molecular Subtypes). Additionally, consider discussing the broader relevance to immunotherapy beyond PD-1/PD-L1, and how the ICI scores proposed in your study might guide treatment decisions in clinical practice.

Limitations Section:

Please include a dedicated limitations section and elaborate on the following points: The lack of in vivo validation for PPFIA4.

Response: We thank the reviewer for highlighting this important limitation. As our current study primarily employed in vitro assays, we now explicitly acknowledge in the Discussion that in vivo validation of PPFIA4’s functional role and therapeutic relevance remains essential. We have also outlined the need for future studies using animal models or patient-derived samples to confirm our findings. This acknowledgment clarifies the scope of the current study and sets a clear direction for future research.

The potential for dataset bias (GEO vs. TCGA).

Response: We thank the reviewer for pointing out the important issue of potential dataset bias between TCGA and GEO. To minimize such bias, we performed the following measures:

1)Independent Subtype Identification and Validation: The immune subtypes (C1 and C2) were separately identified in the TCGA dataset and independently validated in two GEO datasets (GSE39582 and GSE103479). The consistent results across datasets (confirmed by NMF clustering, t-SNE analysis, and survival differences) demonstrate the robustness and reproducibility of the subtype classification.

2)Standard Preprocessing: All datasets underwent standardized preprocessing procedures, including normalization and log2 transformation. Low-variance genes were removed prior to clustering to reduce noise.

3)Cross-dataset Consistency: In both TCGA and GEO cohorts, the subtypes showed similar immune characteristics, prognostic trends, and immune checkpoint profiles, which reduces the likelihood of dataset-specific artifacts.

4)Additional Clarification: In the revised manuscript (Results section), we have added a brief explanation on how dataset bias was addressed, and emphasized the use of independent validation cohorts to strengthen our conclusions.

These steps enhance confidence in the reliability and transferability of our findings.

The generalizability of your findings across diverse CRC patient populations. Response: We thank the reviewer for raising this important point.. Our study specifically focused on LNM-negative CRC patients to minimize confounding factors associated with advanced disease stages. We acknowledge that the immune

*Powered by Editorial Manager® and ProduXion Manager® from Aries Systems Corporation*

microenvironment may differ significantly in LNM-positive patients, potentially affecting the applicability of our identified immune subtypes. We have addressed this limitation in the revised manuscript and emphasized the need for future studies to validate our findings in broader CRC populations, including those with lymph node involvement.

Language and Formatting:

The manuscript would benefit from a careful grammar and spell check, as well as the correction of typographical errors throughout.

Response: We thank the reviewer for noting the need for language and formatting improvements. In response, we have thoroughly reviewed the manuscript to correct grammatical errors, spelling mistakes, and typographical issues. Additionally, we have ensured consistency in formatting throughout the document. We believe these revisions have enhanced the clarity and readability of the manuscript. ________________________________________

6. PLOS authors have the option to publish the peer review history of their article (what does this mean?). If published, this will include your full peer review and any attached files.

If you choose “no”, your identity will remain anonymous but your review may still be made public.

**Additional Information:**

**Question**

**Financial Disclosure**

Do you want your identity to be public for this peer review? For information about this choice, including consent withdrawal, please see our Privacy Policy.

Reviewer #1: No Reviewer #2: No

**Response**

The author(s) received no specific funding for this work.

Enter a financial disclosure statement that describes the sources of funding for the work included in this submission. Review the [submission guidelines](http://journals.plos.org/plosone/s/submission-guidelines#loc-financial-disclosure-statement) for detailed requirements. View published research articles from [PLOS ONE](https://journals.plos.org/plosone/) for specific examples.

This statement is required for submission and **will appear in the published article** if the submission is accepted. Please make sure it is accurate.

**Funded studies**

Enter a statement with the following details: • Initials of the authors who received each

award

• Grant numbers awarded to each author • The full name of each funder

• URL of each funder website

• Did the sponsors or funders play any role in the study design, data collection and analysis, decision to publish, or preparation of the manuscript?

*Powered by Editorial Manager® and ProduXion Manager® from Aries Systems Corporation*

Did you receive funding for this work?

**Competing Interests**	The authors have declared that no competing interests exist.

Use the instructions below to enter a competing interest statement for this submission. On behalf of all authors, disclose any [competing interests](http://journals.plos.org/plosone/s/competing-interests) that could be perceived to bias this

work—acknowledging all financial support and any other relevant financial or non-financial competing interests.

This statement is required for submission and **will appear in the published article** if the submission is accepted. Please make sure it is accurate and that any funding sources listed in your Funding Information later in the submission form are also declared in your Financial Disclosure statement.

View published research articles from [PLOS ONE](http://journals.plos.org/plosone/) for specific examples.

**NO authors have competing interests**

Enter: The authors have declared that no competing interests exist.

**Authors with competing interests**

Enter competing interest details beginning with this statement:

I have read the journal's policy and the authors of this manuscript have the following competing interests: [insert competing interests here]

* typeset

**Ethics Statement**	N/A

Enter an ethics statement for this submission. This statement is required if the study involved:

*Powered by Editorial Manager® and ProduXion Manager® from Aries Systems Corporation*

• Human participants

• Human specimens or tissue

• Vertebrate animals or cephalopods • Vertebrate embryos or tissues

• Field research

Write "N/A" if the submission does not

require an ethics statement.

General guidance is provided below.

Consult the [submission guidelines](http://journals.plos.org/plosone/s/submission-guidelines#loc-guidelines-for-specific-study-types) for

detailed instructions. **Make sure that all**

**information entered here is included in the**

**Methods section of the manuscript.**

*Powered by Editorial Manager® and ProduXion Manager® from Aries Systems Corporation*

**Format for specific study types**

**Human Subject Research (involving human participants and/or tissue)**

• Give the name of the institutional review board or ethics committee that approved the study

• Include the approval number and/or a statement indicating approval of this research

• Indicate the form of consent obtained (written/oral) or the reason that consent was not obtained (e.g. the data were analyzed anonymously)

**Animal Research (involving vertebrate**

**animals, embryos or tissues)**

• Provide the name of the Institutional Animal Care and Use Committee (IACUC) or other relevant ethics board that reviewed the study protocol, and indicate whether they approved this research or granted a formal waiver of ethical approval

• Include an approval number if one was obtained

• If the study involved non-human primates, add additional details about animal welfare and steps taken to ameliorate suffering

• If anesthesia, euthanasia, or any kind of animal sacrifice is part of the study, include briefly which substances and/or methods were applied

**Field Research**

Include the following details if this study

involves the collection of plant, animal, or

other materials from a natural setting: • Field permit number

• Name of the institution or relevant body that granted permission

**Data Availability**	Yes - all data are fully available without restriction

Authors are required to make all data underlying the findings described fully available, without restriction, and from the time of publication. PLOS allows rare exceptions to address legal and ethical concerns. See the [PLOS Data Policy](http://journals.plos.org/plosone/s/data-availability) and [FAQ](http://journals.plos.org/plosone/s/data-availability#loc-faqs-for-data-policy) for detailed information.

*Powered by Editorial Manager® and ProduXion Manager® from Aries Systems Corporation*

A Data Availability Statement describing where the data can be found is required at submission. Your answers to this question constitute the Data Availability Statement and **will be published in the article**, if accepted.

**Important:** Stating ‘data available on request from the author’ is not sufficient. If your data are only available upon request, select ‘No’ for the first question and explain your exceptional situation in the text box.

Do the authors confirm that all data

underlying the findings described in their

manuscript are fully available without

restriction?

**Describe where the data may be found in** All relevant data are within the manuscript and its Supporting Information files. **full sentences. If you are copying our**

**sample text, replace any instances of XXX with the appropriate details.**

• If the data are **held or will be held in a public repository**, include URLs, accession numbers or DOIs. If this information will only be available after acceptance, indicate this by ticking the box below. For example: All XXX files are available from the XXX database (accession number(s) XXX, XXX.).

• If the data are all contained **within the manuscript and/or Supporting Information files**, enter the following: All relevant data are within the manuscript and its Supporting Information files.

• If neither of these applies but you are able to provide **details of access elsewhere**, with or without limitations, please do so. For example:

Data cannot be shared publicly because of [XXX]. Data are available from the XXX Institutional Data Access / Ethics Committee (contact via XXX) for researchers who meet the criteria for access to confidential data.

The data underlying the results presented in the study are available from (include the name of the third party

*Powered by Editorial Manager® and ProduXion Manager® from Aries Systems Corporation*

and contact information or URL).

• This text is appropriate if the data are owned by a third party and authors do not have permission to share the data.

* typeset

Additional data availability information:	Tick here if the URLs/accession numbers/DOIs will be available only after acceptance of the manuscript for publication so that we can ensure their inclusion before publication.

*Powered by Editorial Manager® and ProduXion Manager® from Aries Systems Corporation*

[Click here to access/download;Manuscript;Manuscript.docx](https://www2.cloud.editorialmanager.com/pone/download.aspx?id=40618743&guid=a436b016-0102-4359-bef2-77b82b807673&scheme=1)

**Immune Subtyping of Lymph Node Metastasis-Negative Colorectal Cancer Reveals Biomarkers for Prognosis and Immunotherapy Response**

WenliangYuan^1*^, Li Liu^1^

1 College of Data Science, Jiaxing University, Jiaxing, Zhejiang, China ^*^Corresponding authors

Email: yuanwl@zjxu.edu.cn

**Abstract**

**Background.**Lymph node metastasis (LNM) is a key prognostic factor in colorectal cancer (CRC), and early lymph node status assessment is crucial for prognosis and immunotherapy decisions. However, the immune characteristics of LNM-negative CRC remain poorly understood.

**Methods.**Using machine learning algorithms, we identified and analyzed two immune subtypes (C1 and C2) in 244 LNM-negative CRC samples, with validation in 458 additional samples, and evaluated theirimmune characteristics and functional pathways. **Results.**Subtype C1 exhibited high immune scores and responsiveness to immune checkpoint blockade, while subtype C2 showed a favorable prognosis and increased immune cell infiltration, indicating it represents an earlier CRC stage. Experimental validation revealed that *PPFIA4* knockdown in C1 significantly suppressed CRC cell proliferation and migration.

**Conclusions.**These findings provide insights into personalized immunotherapy strategies for early-stage CRC patients and have potential clinical application value. Subjects Bioinformatics, Genomics, Oncology

Keywords CRC; immunotherapy; LNM-negative; PPFIA4

**Introduction**

Colorectal cancer (CRC) is the third most prevalent malignancy globally, comprising approximately 10.2% of cancer cases and causing a 9.2% mortality rate [1]. Metastasis occurs in 50–60% of CRC patients, frequently resulting in poor surgical outcomes and diminished survival due to disease recurrence [2, 3]. Translymphatic spread is a common metastatic pathway, significantlyimpacting long-term survival [4]. Therefore, early evaluation of lymph node status is crucial for accurate prognosis and treatment planning. However, the TNM staging, a primary prognostic tool for CRC, often lacks granularity, as patients within the same stage can exhibit substantial variability in survival outcomes post-surgery [5].

Immunotherapy, especially PD-1/PD-L1 blockade, has become a pivotal strategy in cancer treatment by enhancing anti-tumor immune responses [6]. Immune cells play a vital rolein CRC progression, influencingboth tumor initiation and therapeuticefficacy [7].The tumor microenvironment (TME), especially interactions between immune and CRC cells, shapes tumor behavior and modulates treatment response [8, 9]. For example, M2-polarized macrophages contribute to chemotherapy resistance and promote CRC cell migration [10]. However, immunotherapy is typically effective only in patients with specific tumor features like defective mismatch repair (dMMR) or high microsatellite instability (MSI) [11]. While recent classifications of MSI-high CRC have identified distinct immune subtypes [12] , the immunological landscape of lymph node metastasis-negative (LNM-negative) CRC remains largely unexplored. A comprehensive classification system for LNM-negative CRC could facilitate the identification of biomarkers for personalized immunotherapy.

In this study, we utilize machine learning algorithms to identify novel immune subtypes in LNM-negative CRC using large-scale datasets. These subtypes are characterized by their prognostic features, immune profiles, transcriptomic patterns, clinical traits, immune cell infiltration, and genetic mutations. We also introduce an immune checkpoint inhibition (ICI) score to assess immune states and investigate the functional role of *PPFIA4* in CRC cell proliferation and migration. Our findings provide novel insights into the refined classification of CRC immune subtypes and highlight potential biomarkers for optimizing immunotherapy, particularly in LNM-negative patients.

**Materials and methods**

**Data sources and preprocessing**

Colorectal cancer (CRC) data were sourced from The Cancer Genome Atlas (TCGA; 244 samples) and Gene Expression Omnibus (GEO; 302 from GSE39582 and 156 from GSE103479). After excluding genes with low median absolute deviation (MAD), a univariate Cox proportional hazards model was applied to link gene expression with overall survival. Focusing on 2,995 immune-related genes [13], we employed non-negative matrix factorization (NMF) for clustering, as it is well-suited for high-dimensional transcriptomic data and has been widely used to identify biologically interpretable cancer subtypes. The clustering results were validated using t-distributed stochastic neighbor embedding (t-SNE). Genes positivelyassociated with clusters were classified as ICI feature A, while the remaining were designated as feature B. The Boruta algorithm [14] further refined these features, and principal component analysis (PCA) was applied to derive the first principal component from each set, summarizing key dimensions into interpretable components to construct the ICI score (ICI score =

PC1A − PC1B).

**Analysis of Biological Functions and Immune Landscape of**

**CRC Subtypes**

Gene set variation analysis (GSVA) was used to evaluate sample-pathway relationships by calculating enrichment scores with 29 CRC-related gene signatures across four categories: Signatures, Canonical, Immune, and Metabolism [15] Gene Set Enrichment Analysis (GSEA) was then performed to assess statistical significance between high and low ICI score groups [16]. To characterize CRC subclasses, immune infiltration was estimated using both the MCP-counter and single-sample GSEA(ssGSEA), which calculated enrichment scores for 13 immune cell groups [17]. Differentially expressed mRNAs between LN-negative CRC subclasses were identified using edgeR with a criterion of |log2 fold change|>= 1.5 and FDR <0.01 [18]. Immuneand stromal scores were computed using the ESTIMATE algorithm [19].

**Cell Culture, Transfection, and qPCR Analysis**

Human colorectal cancer cell lines SW480 (FH0022) and HCT-116 (FH0027) were obtained from Fuheng Biotechnology (Shanghai, China). SW480 cells were cultured in L15 medium, and HCT-116 in McCoy's 5A, both supplemented with 10% FBS and 1% penicillin/streptomycin. Cells were seeded in 6-well plates and transfected at 70-80% confluence with Lipofectamine 3000 using si-NC or si-*PPFIA4*. After 48 hours, RNA was extracted for qPCR analysis. RNA was reverse-transcribed and analyzed by qPCR on an X960 Real-time Thermal Cycler using SYBR Green, with GAPDH as the reference gene. Gene expression was quantified by the 2−ΔΔCq method, using primers for	*PPFIA4*	(F:	5’-CTCTGCGGATGTTGTCTCCC-3’,	R:	5’-ATGCTGCCACTGGTTACACG-3’)	and	*GAPDH*	(F:	5’-GGAGCGAGATCCCTCCAAAAT-3’, R: 5’-GGCTGTTGTCATACTTCTCATGG-3’).

**Protein**	**Analysis,**	**Cell**	**Proliferation,**	**and**	**Migration**

**Evaluation**

Proteins were extracted and quantified using a BCA assay, separated by SDS-PAGE, and transferred to PVDF membranes. After blocking and incubating with primary antibodies against liprin α4 and GAPDH, membranes were processed with secondary antibodies, and signals detected using an ECL kit. Cell proliferation was assessed by MTT assay on days 1, 3, 5, and 7, measuring OD at 570 nm. Significant proliferation

was observed in SW480 on days 5 and 7 (P < 0.05) and in HCT-116 from day 3 (P < 0.01). In the scratch assay, confluent cells were scratched, and wound healing was monitored at 0, 24, and 48 hours. Wound healing was quantified by measuring wound area in three random fields per well.

**Statistical analyses**

Analyses were conducted using R (version 3.5.0), with chi-square tests for immune scores, clinical data, and mutation rates. The univariate Cox model was used to identify survival-associated genes, and Kaplan-Meier curves were generated with P < 0.05 considered significant.

**Results**

**Identification and Validation of Two Molecular Subtypes in**

**LNM-negative CRC**

We selected 109 candidate genes from immune-related and CRC survival-associated genes for non-negative matrix factorization (NMF) analysis (S1 Table). NMF consensus clustering of the TCGA dataset identified two distinct molecular subtypes (𝑘=2) with clear boundaries in the consensus matrix (Fig. 1A). To ensure comparability across datasets and minimize potential batch effects, we performed standardized data preprocessing before conducting cross-cohort validation. Chi-square filtering revealed 80 genes significantly associated with the clustering results (S1 Table). Independent analyses of GEO datasets (GSE39582 and GSE103479) confirmed two molecular subclasses in LNM-negative CRC (Fig. 1D, S1A Fig.). T-SNE analysis further supported the consistency of the clustering across both datasets (Figs. 1B and1E). Prognostic differences were observed in both TCGA and GEO datasets (Figs. 1C and 1F), with significant differences in "Pathologic stage" and "Pathologic M" proportions between subclasses (Table 1), and in "Pathologic T" and "Vital status" in GSE39582. Together, these results demonstrate that the identified molecular subtypes are robust, reproducible across independent datasets, and are associated with distinct gene expression profiles and prognostic outcomes in LNM-negative CRC.

**Fig 1. Use non-negative matrix factorization (NMF) to identify subclasses in LNM negative CRC.** (A) Use immune-related genes to perform NMF clustering on the TCGA. Cophenetic correlation coefficient for k = 2-6 is shown. (B) T-SNE analysis on the TCGA supports dividing it into two subclasses. (C) Overall survival (OS) analysis of the two subclasses (C1 and C2) in TCGA. (D) Use immune-related genes to perform

NMF clustering on the GSE39582. (E) T-SNE analysis on the GSE39582 supports dividing it into two subclasses. (F) OS analysis of the two subclasses (C1 and C2) in GSE39582, with statistical significance assessed by log-rank test

**Fig S1. Validation of molecular subclasses in LNM-negative CRC using the GSE103479 dataset.** (A) Use immune-related genes to perform NMF clustering on the GSE103479. Cophenetic correlation coefficient for k = 2-6 is shown. (B) Overall survival (OS) analysis of the two subclasses (C1 and C2) in GSE103479, with statistical significance assessed by log-rank test

Table 1. Clinical Characteristics of patients with distinct classification in TCGA and

GSE39582

**LN-negative**

**Age(years)(%)**

TCGA

C1	C2	P	C1 81	163	207

0.071

GSE39582

C2	P 95

0.265

> 68

<= 68

**Gender(%)**

Male

Female

**Pathologic stage(%)**

Stage I

Stage II

Stage III

Stage IV

**Pathologic T(%)**

T1-T2

T3-T4

**Pathologic M(%)**

M0

M1

MX

**Vital status(%)**

Alive

Dead

36(44.4)

45(55.6)

43(53.1)

38(46.9)

17(21.0)

61(75.3)

0(0.0)

1(1.2)

18(22.2)

63(77.8)

71(87.7)

1(1.2)

9(11.1)

68(84.0)

13(16.0)

82(50.3)

61(49.7)

0.494

95(58.3)

68(41.7)

**0.027**

56(34.4)

95(58.3)

0(0.0)

7(4.3)

0.074

57(35.1)

106(64.9)

**0.001**

141(86.5)

7(4.3)

15(9.2)

0.136

148(91.8)

15(9.2)

92(44.4)

115(55.6)

116(56.0)

91(44.0)

21(10.1)

174(84.1)

0(0.0)

12(5.8)

20(9.6)

186(90.4)

194(93.7)

13(6.3)

0(0.0)

152(73.4)

55(26.6)

49(51.6)

46(48.4)

0.803

55(57.9)

40(42.1)

**0.019**

16(16.8)

79(83.2)

0(0.0)

0(0.0)

**0.039**

18(18.9)

77(81.1)

**0.016**

94(98.9)

0(0.0)

1(1.1)

**0.001**

85(89.4)

10(10.6)

**Correlation of the LN-negative CRC Subtypes With Immune-**

**Associated Signatures**

To characterize the immune features of the two LN-negative CRC subtypes, we performed differential gene expression analysis and identified 160 subtype-specific signature genes(S2 Table). Gene ontology enrichment revealed significant involvement

of these genes in immune-related processes, including T cell and lymphocyte proliferation (S3 Table), and pathways such as Th17 cell differentiation (S4 Table). GSVAanalysis of 29 CRC-relevant immune signatures revealed significant differences in canonical pathways like MAPK, as well as immune pathways such as immune response, PD1 activation, and complement activation in C2 (Fig. 2A, S5 Table). Scoring analysis showed that C2 had lower immune, stromal, and cytolytic scores than C1, with no significant difference in proliferation scores (Figs. 2B-2E), suggesting distinct immune activity profiles between the subtypes.

**Fig 2. Association between immune-associated signatures and LN-negative CRC subclasses.** (A) Heatmap of specific immune-associated signatures. Boxplots for immune score (B), stromal score (C), cytolytic score (D), and proliferation score (E) in LN-negative CRC subclasses (C1 and C2). Statistical differences were assessed using the Kruskal–Wallis test; asterisks indicate significance levels (ns = not significant, * P < 0.05, **** P < 0.0001).

**Immune Cell Infiltration and Immune Checkpoint Profiles in**

**LN-negative CRC Subtypes**

Given the observed immune score differences, we further evaluated immune cell infiltration using MCP counter and ssGSEA, calculating the abundance of 13 immune cell types in each subtype (Fig. 3A). Consistent with C1’s stromal enrichment, 12 immune cell types (excludingneutrophils) weremore abundant in C1, includingTcells, CD8^+^ Tcells, cytotoxic lymphocytes, and various myeloid cells (Fig. 3B, S6 Table). To investigate the immune checkpoint landscape, we examined the expression of 19 clinically targetable immune checkpoint genes. C1 showed higher expression of 17 checkpoint genes compared to C2, with TNFSF14 and TNFSF15 as exceptions(Fig. 3C, S7 Table). Furthermore, TIDE algorithm predictions indicated higher T cell exhaustion in C1, implyingthat patients in this subclass maybenefit more from immune checkpoint inhibition therapy (Fig. 3D, S8 Table).

**Fig 3. Correlation between LN-negative CRC subclasses and immune infiltration.** (A) Heatmap of immune and stromal cell populations. (B) Boxplot of immune and stromal cell population abundance in subclasses C1 and C2. (C) Expression levels of 19 immune checkpoint genes across subclasses. (D) Boxplot of TIDE scores forthe two subclasses. Statistical differences wereassessed usingthe Kruskal–Wallis test; asterisks indicate significance levels (ns = not significant, * P < 0.05, ** P < 0.01, **** P < 0.0001).

**Genomic Mutations and Immune Response Differences**

**Between LN-negative CRC Subtypes**

Somatic mutations are a hallmark of malignancy. We identified significant mutation differences between LNM-negative CRC subtypes (Fig. 4A). Both subtypes showed high mutation frequencies in genes likeAPC, TTN, and TP53, but with differing rates. *KRAS* mutations were detectedin 67% of C2 samples but only 39% of C1, and were absent from C1’s top 10 mutated genes. Similarly, *BRAF* mutations were more frequent in C2 (S9 Table), indicating distinct genomic profiles. TMB, associated with a stronger immune response, was higher in C1 (Fig. 4B). In addition, we also found that C1 had a greater neoantigen burden than C2 (Fig. 4D), with C2 showing significantly lower neoantigen and neoantigen origin protein burdens (Fig. 4C). CNV analysis confirmed these differences (Fig. 4E), suggesting that C1’s higher mutation load increases neoantigen production, activating more T cells and enhancing immune response.

**Fig 4 Association between LN-negative CRC subclasses and mutations, TMB, neoantigens, and copy number variation (CNV).** (A) Top 10 most mutated genes in subclasses C1 (left) and C2 (right). Boxplots for tumor mutational burden (TMB) (B), neoantigen peptides (C), and neoantigen-related proteins (D) in LN-negative CRC subclasses. (E) CNV distribution in C1 and C2. Statistical differences were assessed using the Kruskal–Wallis test, with asterisks indicating significance levels	(**** P < 0.0001).

**Prognostic Value of ICI Scores and Pathway Enrichment in**

**LNM-negative CRC**

To quantify the immune contexture of LN-negative CRC, we constructed ICI scores by correlating SGRCs with molecular subtypes in the TCGA and GSE39582 cohorts. ICI scores were computed based on the expression of signature gene sets A and B. Significant differences in ICIscores wereobserved between thetwo subtypes(Figs. 5A-5B, S10 Table). Using the optimal cut-off, patients were divided into high and low ICI score groups. Kaplan-Meier survival analysis showed better overall survival for high ICI score patients (Figs. 5C-5D). GSEA revealed limited pathway enrichment in the high ICI score group, with only 4 pathways in TCGA (Fig. 5E) and the RIBOSOME pathway in GSE39582 (Fig. 5F). In contrast, the low ICI score group showed enrichment in multiple immune-related pathways, such as B cell receptor signaling and intestinal immune network for IgA production.

**Fig 5 Construction of the ICI score.** (A, B) Boxplots of ICI scores for the two subcategories in TCGA and GSE39582 datasets. (C, D) Survival analysis for high and

low ICI subgroups in TCGAand GSE39582. (E, F) Enrichment plots for high and low ICI groups in TCGAand GSE39582, with the upper and lower halves of the horizontal axis representing the low and high ICI score subgroups, respectively. Statistical differences were assessed using the Kruskal–Wallis test, with asterisks indicating significance levels (**** P < 0.0001).

**Expression and Functional Analysis of *PPFIA4* in CRC**

**Subtypes**

We observed significant differences in PPFIA4 expression between C1 and C2, suggesting its involvement in subtype-specific immune responses. PPFIA4 inhibition via siRNA transfection led to a marked reduction in its mRNA (Fig. 6A) and protein levels (Fig. 6B). Proliferation (MTT) and wound healing assays showed that PPFIA4 inhibition significantly reduced cell proliferation and migration (Fig. 6C, 6D). Collectively, these findings indicate that PPFIA4 plays a key role in colorectal cancer cell proliferation and migration, making it a potential therapeutic target.

**Fig 6. PPFIA4 Knockdown Suppresses Expression, Proliferation, and Migration in Colorectal Cancer Cells.** (A) qPCR analysis showing a significant reduction in PPFIA4 mRNA expression in the si-PPFIA4 group.(B) Western blot (WB) validation of decreased liprin α4 protein expression in the si-PPFIA4 group, confirming transfection efficiency.(C) MTT assay demonstrating a marked reduction in cell proliferation upon PPFIA4 knockdown.(D) Wound healing assay results indicating a significant impairment in cell migration at 0, 24, and 48 hours post-scratch following PPFIA4 knockdown. (* P < 0.05, ** P < 0.01,**** P < 0.0001).

**4. Discussion**

Lymph node metastasis (LNM) is a critical prognostic factor in CRC, influencing clinical treatment strategies, particularly in rectal cancer. Despite advances, the 5-year survival rate post-surgery remains suboptimal [20]. Recent studies emphasize the potential of immunotherapy, with PD-L1/PD-1 interactions modulating T cell exhaustion [21]. In this study, we identified two distinct LNM-negative CRC subtypes (C1 and C2) based on 2,995 immune-related genes and machine learning, and demonstrated their prognostic, immune, and mutational differences. To further stratify patients, we constructed an immune checkpoint inhibition (ICI) score, serving as a prognostic biomarker and a predictor of immunotherapy response. Notably, higher ICI scores were associated with lower immune and stromal scores, whereas lower ICI scores correlated with increased immune infiltration, suggesting the utility of our

subtype classification in guiding immunotherapy strategies. Subtype C1 exhibited high immune scores and responsiveness to immune checkpoint inhibitors, while C2, with favorable prognosis and high ICI scores, may represent an earlier stage of CRC. This immune-based subtyping approach differs from broader classifications like the Consensus Molecular Subtypes (CMS) by specificallytargeting LNM-negative CRC to guide immunotherapy decisions. To reduce heterogeneity, our analysis was limited to LNM-negative cases; however, immune profiles may differ in LNM-positive patients. Further studies are needed to assess the generalizability of these subtypes across CRC stages.

Activation of the MAPK pathway through *KRAS* influences cell proliferation and differentiation, potentially preventing tumorigenesis [22]. CRCs with BRAF mutations is associated with lymphocyte infiltration and immune response activation [23], with studies indicating a positive correlation between PD-L1 expression and BRAF mutations. High CD8+ tumor-infiltrating lymphocytes observed in BRAF-mutant CRC suggest that these patients may respond well to immunotherapy [24]. Although *KRAS* mutations are generally unrelated to LNM status or tumor characteristics [4, 8], our study demonstrates a shift in *KRAS* mutation rates across the two LNM-negative subtypes, potentially offering survival benefits to CRC patients with BRAF mutations.

To identify diagnostic biomarkers for CRC subtypes, We found that PPFIA4 was significantly upregulated in subtype C1 compared to C2. Research on PPFIA4 in CRC is still in its early stages, and it may be associated with aberrant metabolic processes, positioningitas atarget genein cancer glycolysis [25]. Previousstudies haveconfirmed that *PPFIA4* serves as a crucial molecular mechanism mediating intestinal carcinogenesis by influencing immune cell expression [26]. In our study, elevated *PPFIA4* expression was associated with poorer overall survival, while its knockdown markedly inhibited CRC cell proliferation and migration, indicating a potential role in tumor progression. Beyond its biological function, *PPFIA4* holds promise as a clinically actionable biomarker, potentially serving both diagnostic and therapeutic purposes. Its subtype-specificexpression profilecould help distinguish moreaggressive immune subtypes in early-stage CRC, and its inhibition may offer a new target for anti-tumor intervention. However, as our findings are based solely on in vitro data, further validation through in vivo studies using patient tissues or animal models is necessary to confirm its clinical utility.

In conclusion, we conducted an in-depth analysis of the immune signature of LNM-negative CRC, especially regarding the relationship between immune subtypes and immunotherapy response. These findings enhance our understanding of CRC progression and may inform personalized strategies for cancer immunotherapy.

**Competing interests**

The author declares that he has no competing interests.

**Ethics approval and consent to participate** Not applicable.

**Patient consent for publication** Not applicable.

**References**

1.	Bray F, Laversanne M, Sung H, Ferlay J, Siegel RL, Soerjomataram I, et al. Global cancer statistics 2022: GLOBOCAN estimates of incidence and mortality worldwide for 36 cancers in 185 countries. CA: a cancer journal for clinicians. 2024;74(3):229-63.

2.	Nancy K. The management of resectable and unresectable liver metastases from colorectal cancer. Current Opinion in Oncology. 2010;22(4):364-73.

3.	Lupinacci RM, Andraus W, Haddad LBDP, D ′	Albuquerque LAC, Herman P. Simultaneous laparoscopic resection of primary colorectal cancer and associated liver metastases: a systematic review. Techniques in Coloproctology. 2014;18(2):129-35.

4.	Yuan W, Li X, Liu L, Wei C, Sun D, Peng S, et al. Comprehensive analysis of lncRNA-associated ceRNA network in colorectal cancer. Biochem Biophys Res Commun 2019;508(2):374-9.

5.	Li R, Liu X, Huang X, Zhang D, Chen Z, Zhang J, et al. Single-cell transcriptomic analysis deciphers heterogenous cancer stem-like cells in colorectal cancer and their organ-specific metastasis. Gut. 2024;73(3):470-84.

6.	Dougan M, Dranoff G. Immune Therapy for Cancer. Annual Review of Immunology. 2009;27(27):83-117.

7.	Al Zein M, Boukhdoud M, Shammaa H, Mouslem H, El Ayoubi LM, Iratni R, et al. Immunotherapy and immunoevasion of colorectal cancer. Drug Discovery Today 2023;28(9):103669.

8.	Markman JL, Shiao SL. Impact of the immune system and immunotherapy in colorectal cancer. Journal of gastrointestinal oncology. 2015;6(2):208-23.

9.	Yuan W, Cai W, Huang X, Peng S. Prognostic value of immune scores in the microenvironment of colorectal cancer. Oncology Letters. 2020;20(5):1-.

10. Zhu X, Liang R, Lan T, Ding D, Huang S, Shao J, et al. Tumor-associated macrophage-specific CD155 contributes to M2-phenotype transition, immunosuppression, and tumor progression in colorectal cancer. Journal for immunotherapy of cancer. 2022;10(9). Epub 2022/09/15. doi: 10.1136/jitc-2021-004219. PubMed PMID: 36104099; PubMed Central PMCID: PMCPmc9476138.

11. Overman MJ, Mcdermott R, Leach JL, Lonardi S, André T. Nivolumab in patients with metastatic DNA mismatch repair-deficient or microsatellite instability-high colorectal cancer (CheckMate 142): an open-label, multicentre, phase 2 study. Lancet Oncology. 2017;18(9):1182-91.

12. Diaz LA, Jr., Shiu KK, Kim TW, Jensen BV, Jensen LH, Punt C, et al. Pembrolizumab versus chemotherapy for microsatellite instability-high or mismatch repair-deficient metastatic colorectal cancer (KEYNOTE-177): final analysis of a randomised, open-label, phase 3 study. The Lancet Oncology.

2022;23(5):659-70. Epub 2022/04/16. doi: 10.1016/s1470-2045(22)00197-8. PubMed PMID: 35427471; PubMed Central PMCID: PMCPmc9533375.

13. Thorsson V, Gibbs DL, Brown SD, Wolf D, Bortone DS, Yang T-HO, et al. The immune landscape of cancer. Immunity. 2018;48(4):812-30. e14.

14. Kursa MB, Rudnicki WR. Feature Selection with Boruta Package. Journal of Statistical Software. 2010;36(11):1-13.

15. Hnzelmann S, Castelo R, Guinney J. GSVA: gene set variation analysis for microarray and RNA-Seq data. BMC Bioinf 2013;14(1):7-.

16. Subramanian A, Tamayo P, Mootha VK, Mukherjee S, Ebert BL, Gillette MA, et al. Gene set enrichment analysis: a knowledge-based approach for interpreting genome-wide expression profiles. Proceedings of the National Academy of Sciences. 2005;102(43):15545-50.

17. Becht E, Giraldo NA, Lacroix L, Buttard… B. Estimating the population abundance of tissue-infiltrating immune and stromal cell populations using gene expression. Genome Biology. 2016;17(1):218.

18. Robinson MD, McCarthy DJ, Smyth GK. edgeR: a Bioconductor package for differential expression analysis of digital gene expression data. Bioinformatics. 2010;26(1):139-40.

19. Yoshihara K, Shahmoradgoli M, Martínez E, Vegesna R, Kim H, Torresgarcia W, et al. Inferring tumour purity and stromal and immune cell admixture from expression data. Nature Communications. 2013;4:2612.

20. Yu B, Kang J, Lei H, Li Z, Yang H, Zhang M. Immunotherapy for colorectal cancer. Front Immunol. 2024;15:1433315. Epub 2024/09/06. doi: 10.3389/fimmu.2024.1433315. PubMed PMID: 39238638; PubMed Central PMCID: PMCPmc11375682.

21. Wang X, Li M. Correlate tumor mutation burden with immune signatures in human cancers. BMC Immunology. 2019;20(1):4.

22. Sorokin AV, Kanikarla Marie P, Bitner L, Syed M, Woods M, Manyam G, et al. Targeting RAS Mutant Colorectal Cancer with Dual Inhibition of MEK and CDK4/6. Cancer Res. 2022;82(18):3335-44. Epub 2022/08/02. doi: 10.1158/0008-5472.can-22-0198. PubMed PMID: 35913398; PubMed Central PMCID: PMCPmc9478530.

23. Guinney J, Dienstmann R, Wang X, Reyniès A, Tejpar S. The consensus molecular subtypes of colorectal cancer. Nat Med 2015;21(11):1350-6.

24. Du W, Frankel TL, Green M, Zou W. IFNγ signaling integrity in colorectal cancer immunity and immunotherapy. Cellular & molecular immunology. 2022;19(1):23-32. Epub 2021/08/14. doi: 10.1038/s41423-021-00735-3. PubMed PMID: 34385592; PubMed Central PMCID: PMCPmc8752802. 25. Pate KT, Stringari C, Sprowl‐ Tanio S, Wang K, TeSlaa T, Hoverter NP, et al. Wnt signaling directs a metabolicprogram of glycolysisand angiogenesisin colon cancer. TheEMBO journal. 2014;33(13):1454-73.

26. Zheng FX, Yang CR, Sun FY, Zhang YZ, Wang YL, Li XZ, et al. Enterotoxin‐ related genes PPFIA4 and SCN3B promote colorectal cancer development and progression. J Biochem Mol Toxicol 2024;38(6):e23746.

**Supporting information**

**S1 Table. The 109 immune associated genes used for classification. S2 Table. The result of differential expression analysis.**

**S3 Table. Functional enrichment analyses of subclass specific genes. S4 Table. Pathway enrichment analysis of two CRC subclasses.**

**S5 Table. P values for gene set mRNA enrichment analysis. S6 Table. The abundances of 13 immune-related cells.**

**S7 Table. Expression profiles of 19 potential targeted immune checkpoint genes in two subtypes.**

**S8 Table. TIDE value of immune checkpoint suppression therapy for each sample. S9 Table. Genetic mutation analysis of GSE39582 dataset.**

**S10 Table. ICI scores for each sample in TCGA and GSE39582.**

[C](https://www2.cloud.editorialmanager.com/pone/download.aspx?id=40618733&guid=0ed163dc-c272-4b4e-8079-bd79a5ac9c50&scheme=1)over Letter	Click here to access/download;Figure;fig 1-01.tif
[C](https://www2.cloud.editorialmanager.com/pone/download.aspx?id=40618734&guid=a74f3db6-1ca9-454c-8da5-a8b18ac020b1&scheme=1)over Letter	Click here to access/download;Figure;fig 2-01.tif
[C](https://www2.cloud.editorialmanager.com/pone/download.aspx?id=40618735&guid=259587fa-55ed-4c45-9a7f-47bea2c6f7dc&scheme=1)over Letter	Click here to access/download;Figure;fig 3-01.tif
[C](https://www2.cloud.editorialmanager.com/pone/download.aspx?id=40618736&guid=0c0a1df9-5e36-42ba-ae47-2622cef84c26&scheme=1)over Letter	Click here to access/download;Figure;fig 4-01.tif
[C](https://www2.cloud.editorialmanager.com/pone/download.aspx?id=40618737&guid=7c41c954-b864-4436-8e9e-3420a721590d&scheme=1)over Letter	Click here to access/download;Figure;fig 5-01.tif
[C](https://www2.cloud.editorialmanager.com/pone/download.aspx?id=40618738&guid=8122cca5-7da2-43d9-b3b2-f1cf384cda5f&scheme=1)over Letter	Click here to access/download;Figure;fig6-01.tif
[C](https://www2.cloud.editorialmanager.com/pone/download.aspx?id=40618739&guid=50d4ca87-dcf5-4eb5-8b91-4236f728cc09&scheme=1)over Letter	Click here to access/download;Figure;fig s1-01.tif

Cover Letter

[Click here to access/download](https://www2.cloud.editorialmanager.com/pone/download.aspx?id=40618740&guid=792222d7-a4dd-4f54-8196-ae3848c08f76&scheme=1) [**Supporting Information**](https://www2.cloud.editorialmanager.com/pone/download.aspx?id=40618740&guid=792222d7-a4dd-4f54-8196-ae3848c08f76&scheme=1) [SupplementaryTable-S1-S10.xlsx](https://www2.cloud.editorialmanager.com/pone/download.aspx?id=40618740&guid=792222d7-a4dd-4f54-8196-ae3848c08f76&scheme=1)

Manuscript

**Immune Subtyping of Lymph Node Metastasis-Negative Colorectal Cancer Reveals Biomarkers for Prognosis and Immunotherapy Response**

Wenliang Yuan^1*^, Li Liu^1^

1 College of Data Science, Jiaxing University, Jiaxing, Zhejiang, China ^*^Corresponding authors

Email: yuanwl@zjxu.edu.cn

**Abstract**

**Background.**Lymph node metastasis (LNM) is a key prognostic factor in colorectal cancer (CRC), and early lymph node status assessment is crucial for prognosis and immunotherapy decisions. However, the immune characteristics of LNM-negative CRC remain poorly understood.

**Methods.**Using machine learning algorithms, we identified and analyzed two immune subtypes (C1 and C2) in 244 LNM-negative CRC samples, with validation in 458 additional samples,andevaluated their immune characteristicsandfunctional pathways. **Results.**Subtype C1 exhibited high immune scores and responsiveness to immune checkpoint blockade, while subtype C2 showed a favorable prognosis and increased immune cell infiltration, indicating it represents an earlier CRC stage. Experimental validation revealed that *PPFIA4* knockdown in C1 significantly suppressed CRC cell proliferation and migration.

**Conclusions.**These findings provide insights into personalized immunotherapy strategies for early-stage CRC patients and have potential clinical application value. Subjects Bioinformatics, Genomics, Oncology

Keywords CRC; immunotherapy; LNM-negative; PPFIA4

**Introduction**

Colorectal cancer (CRC) is the third most prevalent malignancy globally, comprising approximately 10.2% of cancer cases and causing a 9.2% mortality rate [1]. Metastasis occurs in 50–60% of CRC patients, frequently resulting in poor surgical outcomes and diminished survival due to disease recurrence~~often leading to suboptimal surgical~~ ~~outcomes and reduced survival due to recurrence~~ [2, 3]. Translymphatic spread is a common metastatic pathway, significantlyimpacting long-term survival [4]. Therefore, early evaluation of lymph node status is crucial for accurate prognosis and treatment planning. However, the TNM staging, a primary prognostic tool for CRC, often lacks granularit~~yprecision,~~aspatientswithin thesame stagecanexhibitsubstantial variability in survival outcomes post-surgery [5].

Immunotherapy, especially PD-1/PD-L1 blockade, has become a pivotal strategy in cancer treatment by enhancing anti-tumor immune response~~shas emerged as a key~~

~~cancer treatment by boosting cellular immune responses~~ [6]. Immune cells play a vital

**Formatted:** Font: (Default) Times New Roman, 12 pt

**Formatted:** Font: (Default) Times New Roman, 12 pt, Not Bold, Not Italic

role in CRC progression, influencing both tumor initiation and therapeutic efficac~~yaffecting	tumor	initiation	and	therapy	outcomes~~	[7]. The	tumor microenvironment (TME), especially interactions between immune and CRC cells, shapes tumor behavior and modulates treatment response~~The tumor microenvironment~~ ~~(TME) interactions between immune and CRC cells influence tumor behavior and~~ ~~treatment efficacy~~ [8, 9]. For example, M2-polarized macrophages contribute to chemotherapy resistance and promote CRC cell migration~~M2 macrophages enhance~~ ~~chemotherapy resistance and CRC cell migration~~ [10]. However, immunotherapy is typically effective only in patients with specific tumor features like defective mismatch repair (dMMR) or high microsatellite instability (MSI) [11]. While recent classifications of MSI-high CRC have identified distinct immune subtypes [12] , the immunological landscape of lymph node metastasis-negative (LNM-negative) CRC remains largely unexplored. A comprehensive~~robust~~ classification system for LNM-negative CRC could facilitate the identification of biomarkers for personalized immunotherapy.

In this study, we utilize machine learning algorithms to identify novel immune subtypes in LNM-negative CRC using large-scale~~from large~~ datasets. These subtypes are characterized bytheir prognostic features, immune profiles, transcriptomic patterns, clinical traits, immune cell infiltration, and genetic mutations. We also introduce an immune checkpoint inhibition (ICI) score to assess immune states and investigate the

functional role of *PPFIA4* in CRC cell proliferation and migration~~explore~~ *~~PPFIA4~~*~~'s~~	**Formatted:** Font: Italic ~~role in CRC cell proliferation and migration. Our results provide new insights into the~~

~~precise classification of CRC immune subtypes and offer potential biomarkers for the~~ ~~selection of immunotherapy, especiallyin LNM-negativepatients.~~Our findingsprovide novel insights into the refined classification of CRC immune subtypes and highlight potential biomarkers for optimizing immunotherapy, particularly in LNM-negative patients.

**Materials and methods**

**Data sources and preprocessing**

Colorectal cancer (CRC) data were sourced from The Cancer Genome Atlas (TCGA; 244 samples) and Gene Expression Omnibus(GEO; 302 from GSE39582 and 156 from GSE103479). After excluding genes with low median absolute deviation (MAD), a univariate Cox proportional hazards model was applied to link gene expression with overall survival. Focusing on 2,995 immune-related genes [13], ~~we employed non-~~

~~negative matrix factorization (NMF)for clustering, validated byt-distributed stochastic~~ ~~neighbor embedding (t-SNE). w~~e employed non-negative matrix factorization (NMF) for clustering, as it is well-suited for high-dimensional transcriptomic data and has been widelyusedtoidentifybiologicallyinterpretablecancersubtypes. Theclustering results were validated using t-distributed stochastic neighbor embedding (t-SNE). Genes positivelyassociated with clusters were classified as ICI featureA, while the remaining were designated as feature B. The Boruta algorithm [14] further refined these features, ~~and principal component analysis (PCA) extracted the first principal component (PC1)~~ ~~for each. The ICI score for each patient was calculated as ICI score = PC1A-~~PC1~~B.~~ and principal componentanalysis (PCA) wasapplied to derive the first principalcomponent from each set, summarizing key dimensions into interpretable components to construct

the ICI score (ICI score = PC1A − PC1B).

**Formatted:** Subscript

**Formatted:** Subscript

**Analysis of Biological Functions and Immune Landscape of**

**CRC Subtypes**

Gene set variation analysis(GSVA) was used to evaluate sample-pathway relationships by calculating enrichment scores with 29 CRC-related gene signatures across four categories: Signatures, Canonical, Immune, and Metabolism [15] Gene Set Enrichment Analysis (GSEA) was then performed to assess statistical significance between high and low ICI score groups [16]. To characterize CRC subclasses, immune infiltration was estimated using both the MCP-counter and single-sample GSEA(ssGSEA), which calculated enrichment scores for 13 immune cell groups [17]. Differentially expressed mRNAs between LN-negative CRC subclasses were identified using edgeR with a criterionof |log2 fold change|>= 1.5 andFDR < 0.01[18].Immuneandstromal scores were computed using the ESTIMATE algorithm [19].

**Cell Culture, Transfection, and qPCR Analysis**

Human colorectal cancer cell lines SW480 (FH0022) and HCT-116 (FH0027) were obtained from Fuheng Biotechnology (Shanghai, China). SW480 cells were cultured in L15 medium, and HCT-116 in McCoy's 5A, both supplemented with 10% FBS and 1% penicillin/streptomycin. Cells were seeded in 6-well plates and transfected at 70-80% confluence with Lipofectamine 3000 using si-NC or si-*PPFIA4*. After 48 hours, RNA was extracted for qPCR analysis. RNA was reverse-transcribed and analyzed by qPCR on an X960 Real-time Thermal Cycler using SYBR Green, with GAPDH as the reference gene. Gene expression was quantified by the 2−ΔΔCq method, using primers for	*PPFIA4*	(F:	5’-CTCTGCGGATGTTGTCTCCC-3’,	R:	5’-ATGCTGCCACTGGTTACACG-3’)	and	*GAPDH*	(F:	5’-GGAGCGAGATCCCTCCAAAAT-3’, R: 5’-GGCTGTTGTCATACTTCTCATGG-3’).

**Protein**	**Analysis,**	**Cell**	**Proliferation,**	**and**	**Migration**

**Evaluation**

Proteins were extracted and quantified using a BCA assay, separated by SDS-PAGE, and transferred to PVDF membranes. After blocking and incubating with primary antibodies against liprin α4 and GAPDH, membranes were processed with secondary antibodies, and signals detected using an ECL kit. Cell proliferation was assessed by MTT assay on days 1, 3, 5, and 7, measuring OD at 570 nm. Significant proliferation was observed in SW480 on days 5 and 7 (P < 0.05) and in HCT-116 from day 3 (P < 0.01). In the scratch assay, confluent cells were scratched, and wound healing was monitored at 0, 24, and 48 hours. Wound healing was quantified by measuring wound area in three random fields per well.

**Statistical analyses**

Analyses were conducted using R (version 3.5.0), with chi-square tests for immune scores, clinical data, and mutation rates. The univariate Cox model was used to identify survival-associated genes, and Kaplan-Meier curves were generated with P < 0.05 considered significant.

**Results**

**Identification and Validation of Two Molecular Subtypes in**

**LNM-negative CRC**

We selected 109 candidate genes from immune-related and CRC survival-associated genes for non-negative matrix factorization (NMF) analysis (S1 Table). NMF consensus clustering of the TCGA dataset identified two distinct molecular subtypes (𝑘=2) with clear boundaries in the consensus matrix (Fig. 1A). To ensure comparability across datasets and minimize potential batch effects, we performed standardized data preprocessing before conducting cross-cohort validation. Chi-square filtering revealed 80 genes significantly associated ~~correlated~~ with the clustering results (S1 Table). Independent analyses of GEO datasets (GSE39582 and GSE103479) confirmed two molecular subclasses in LNM-negative CRC (Fig. 1D, S1A Fig.). T-SNE analysis further supported~~corroborated~~ the consistency of the clustering across both datasets (Figs. 1B and~~,~~ 1E). Prognostic differences were observed in both TCGA and GEO datasets (Figs. 1C and~~,~~ 1F), with significant differences in "Pathologic stage" and "Pathologic M" proportions between subclasses (Table 1), and in "Pathologic T" and "Vital status" in GSE39582. Together, these results demonstrate that the identified

molecular subtypes are robust, reproducible across independent datasets, and are associated with distinct gene expression profiles and prognostic outcomes in LNM-negative CRC.

**Fig 1. Use non-negative matrix factorization (NMF) to identify subclasses in LNM negative CRC.** (A) Use immune-related genes to perform NMF clustering on the TCGA. Cophenetic correlation coefficient for k = 2-6 is shown. (B) T-SNE analysis on the TCGA supports dividing it into two subclasses. (C) Overall survival (OS) analysis of the two subclasses (C1 and C2) in TCGA. (D) Use immune-related genes to perform NMF clustering on the GSE39582. (E) T-SNE analysis on the GSE39582 supports dividing it into two subclasses. (F) OS analysis of the two subclasses (C1 and C2) in GSE39582, with statistical significance assessed by log-rank test

**Fig S1. Validation of molecular subclasses in LNM-negative CRC using the GSE103479 dataset.** (A) Use immune-related genes to perform NMF clustering on the GSE103479. Cophenetic correlation coefficient for k = 2-6 is shown. (B) Overall survival (OS)analysisof the two subclasses (C1 andC2) in GSE103479, withstatistical significance assessed by log-rank test

Table 1. Clinical Characteristics of patients with distinct classification in TCGA and

GSE39582

**LN-negative**

**Age(years)(%)**

TCGA

C1	C2	P	C1 81	163	207

0.071

GSE39582

C2	P	**Formatted Table** 95

0.265

> 68

<= 68

**Gender(%)**

Male

Female

**Pathologic stage(%)**

Stage I

Stage II

Stage III

Stage IV

**Pathologic T(%)**

T1-T2

T3-T4

**Pathologic M(%)**

M0

M1

MX

**Vital status(%)**

36(44.4)

45(55.6)

43(53.1)

38(46.9)

17(21.0)

61(75.3)

0(0.0)

1(1.2)

18(22.2)

63(77.8)

71(87.7)

1(1.2)

9(11.1)

82(50.3)

61(49.7)

0.494

95(58.3)

68(41.7)

**0.027**

56(34.4)

95(58.3)

0(0.0)

7(4.3)

0.074

57(35.1)

106(64.9)

**0.001**

141(86.5)

7(4.3)

15(9.2)

0.136

92(44.4)

115(55.6)

116(56.0)

91(44.0)

21(10.1)

174(84.1)

0(0.0)

12(5.8)

20(9.6)

186(90.4)

194(93.7)

13(6.3)

0(0.0)

49(51.6)

46(48.4)

0.803

55(57.9)

40(42.1)

**0.019**

16(16.8)

79(83.2)

0(0.0)

0(0.0)

**0.039**

18(18.9)

77(81.1)

**0.016**

94(98.9)

0(0.0)

1(1.1)

**0.001**

Alive	68(84.0)

Dead	13(16.0)

148(91.8)

15(9.2)

152(73.4)

55(26.6)

85(89.4)

10(10.6)

**Correlation ofthe LN-negative CRC Subtypes With Immune-**

**Associated Signatures**

To characterize the immune features of the two LN-negative CRC subtypes, we performed differential gene expression analysis and identified 160 subtype-specific signature genes~~We conducted differential gene expression analysis to characterize the~~ ~~two LN-negative CRC subclasses, identifying 160 subclass-specific signature genes~~ (S2 Table). Gene ontology enrichment revealed significant involvement of these genes in immune-related processes, including T cell and lymphocyte proliferation (S3 Table), and pathways such as Th17 cell differentiation (S4 Table). GSVA analysis of 29 CRC-relevant immune signatures revealed significant differences in canonical pathways like MAPK, as well as immune pathways such as immune response, PD1 activation, and complement activation in C2 (Fig. 2A, S5 Table). Scoring analysis showed that C2 had lower immune, stromal, and cytolytic scores than C1, with no significant difference in proliferationscores(Figs. 2B-2E), suggestingdistinctimmune activityprofilesbetween the subtypes~~subclasses.~~

**Fig 2. Association between immune-associated signatures and LN-negative CRC subclasses.** (A) Heatmap of specific immune-associated signatures. Boxplots for immune score (B), stromal score (C), cytolytic score (D), and proliferation score (E) in LN-negative CRC subclasses (C1 and C2). Statistical differences were assessed using the Kruskal–Wallis test; asterisks indicate significance levels (ns = not significant, * P < 0.05, **** P < 0.0001).

**Immune Cell Infiltration and Immune Checkpoint Profiles in**

**LN-negative CRC Subtypes**

Given the observed immune score differences, we further evaluated ~~assessed~~ immune cell infiltration using MCP counter and ssGSEA, calculating the abundance of 13 immune cell types in each subtype (Fig. 3A). Consistent with C1’s stromal enrichment, 12 immune cell types (excluding neutrophils) were more abundant in C1, including T

cells, CD8^+^ T cells, cytotoxic lymphocytes, and various myeloid cells (Fig. 3B, S6	**Formatted:** Superscript Table). To investigate the immune checkpoint landscape, we examined the expression

of 19 clinically targetable immune checkpoint genes. C1 showed higher expression of 17 checkpoint genes compared to C2, with TNFSF14 and TNFSF15 as exceptions~~We~~ ~~analyzed 19 targetable immune checkpoint genes from clinical trial drug inhibitors to~~ ~~determine their association with the LN-negative subclasses. Compared with C2, C1~~

~~exhibited higher expression in 17 immune checkpoint genes, with exceptions for~~ ~~TNFSF14 and TNFSF15~~ (Fig. 3C, S7 Table). Furthermore, TIDE algorithm predictions indicated higher T cell exhaustion in C1, implying~~suggesting~~ that patients in this subclass may benefit more from immune checkpoint inhibition therapy (Fig. 3D, S8 Table).

**Fig 3. Correlation between LN-negative CRC subclasses and immune infiltration.** (A) Heatmap of immune and stromal cell populations. (B) Boxplot of immune and stromal cell population abundance in subclasses C1 and C2. (C) Expression levels of 19immune checkpoint genes acrosssubclasses. (D) Boxplot of TIDE scores for thetwo subclasses. Statistical differenceswere assessed usingthe Kruskal–Wallis test; asterisks indicate significance levels (ns = not significant, * P < 0.05, ** P < 0.01, **** P < 0.0001).

**Genomic Mutations and Immune Response Differences**

**Between LN-negative CRC Subtypes**

Somati~~cGene~~ mutations are a hallmark of malignancy. We identified significant mutation differences between LNM-negative CRC subtypes (Fig. 4A). Both subtypes showed high mutation frequencies in genes like APC, TTN, and TP53, but with differing rates. *KRAS* mutations were detecte~~doccurred~~ in 67% of C2 samples but only 39% of C1, and were absent from C1’s top 10 mutated genes~~, while C1 had a 39% rate~~ ~~and no KRAS in its top 10 mutated genes.~~ Similarly, *BRAF* mutations were ~~also~~ more frequent in C2 (S9 Table), indicating distinct genomic profiles. TMB, associated with a stronger immune response, was higher in C1 (Fig. 4B). In addition, ~~W~~we also found that C1 had a greater neoantigen burden than C2 (Fig. 4D), with C2 showing significantly lower neoantigen and neoantigen origin protein burdens (Fig. 4C). CNV analysis confirmed these differences (Fig. 4E), suggesting that C1’s higher mutation load increases neoantigen production, activating more T cells and enhancing immune

response.

**Formatted:** Font: Italic

**Formatted:** Font: Italic

**Fig 4 Association between LN-negative CRC subclasses and mutations, TMB, neoantigens, and copy number variation (CNV).** (A) Top 10 most mutated genes in subclasses C1 (left) and C2 (right). Boxplots for tumor mutational burden (TMB) (B), neoantigen peptides (C), and neoantigen-related proteins (D) in LN-negative CRC subclasses. (E) CNV distribution in C1 and C2. Statistical differences were assessed using the Kruskal–Wallis test, with asterisks indicating significance levels (**** P < 0.0001).

**Prognostic Value of ICI Scores and Pathway Enrichment in**

**LNM-negative CRC**

To quantify the immune contexture of LN-negative CRC, we constructed ICI scores by correlating SGRCs with molecular subtypes in the TCGA and GSE39582 cohorts. ICI scores were computed based on the expression of signature gene sets A and B. Significant differences in ICI scores were observed between the two subtypes~~We~~ ~~constructed ICI scores for LNM-negative CRC patients by correlating SGRCs with~~ ~~subtypes in TCGA and GSE39582, and calculating scores based on signature genes A~~ ~~and B. Significant differences in ICI scores between subtypes were observed (~~Figs. 5A-5B, S10 Table). Using the optimal cut-off, patients were divided into high and low ICI score groups. Kaplan-Meier survival analysis showed better overall survival for high ICI score patients (Figs. 5C-5D). GSEA revealed limited pathway enrichment in the high ICI score group, with only 4 pathways in TCGA (Fig. 5E) and the RIBOSOME pathway in GSE39582 (Fig. 5F). In contrast~~Conversely~~, the low ICI score group showed enrichment in multiple immune-related pathways, such as B cell receptor signaling and intestinal immune network for IgA production.

**Fig 5 Construction of the ICI score.** (A, B) Boxplots of ICI scores for the two subcategories in TCGA and GSE39582 datasets. (C, D) Survival analysis for high and low ICI subgroups in TCGA and GSE39582. (E, F) Enrichment plots for high and low ICI groups in TCGA and GSE39582, with the upper and lower halves of the horizontal axis representing the low and high ICI score subgroups, respectively. Statistical differences were assessed using the Kruskal–Wallis test, with asterisks indicating significance levels (**** P < 0.0001).

**Expression and Functional Analysis of *PPFIA4* in CRC**

**Subtypes**

We observed significant differences in PPFIA4 expression between C1 and C2, suggesting its involvement in subtype-specific immune responses. PPFIA4 inhibition via siRNA transfection led to a marked reduction in its mRNA (Fig. 6A) and protein levels (Fig. 6B). Proliferation (MTT) and wound healing assays showed that PPFIA4 inhibition significantly reduced cell proliferation and migration (Fig. 6C, 6D). Collectively, t~~T~~hese findings indicate that PPFIA4 plays a key role in colorectal cancer

cell proliferation and migration, making it a potential therapeutic target.

**Formatted:** Font: 12 pt

**Formatted:** Font: 12 pt

**Fig 6. PPFIA4 Knockdown Suppresses Expression, Proliferation, and Migration in Colorectal Cancer Cells.** (A) qPCR analysis showing a significant reduction in PPFIA4 mRNA expression in the si-PPFIA4 group.(B) Western blot (WB) validation

of decreased liprin α4 protein expression in the si-PPFIA4 group, confirming transfection efficiency.(C) MTT assay demonstrating a marked reduction in cell proliferation upon PPFIA4 knockdown.(D) Wound healing assay results indicating a significant impairment in cell migration at 0, 24, and 48 hours post-scratch following PPFIA4 knockdown. (* P < 0.05, ** P < 0.01,**** P < 0.0001).

**4. Discussion**

Lymph node metastasis (LNM) is a critical prognostic factor in CRC, influencing clinical treatment strategies, particularly in rectal cancer. Despite advances, the 5-year survival rate post-surgery remains suboptimal [20]. Recent studies emphasize the potential of immunotherapy, with PD-L1/PD-1 interactions modulating T cell exhaustion [21]. In this study, we identified two distinct LNM-negative CRC subtypes (C1 and C2) based on 2,995 immune-related genes and machine learning, and demonstrated their prognostic, immune, and mutational differences. ~~We also~~ ~~constructed immune checkpoint inhibition (ICI) scores as prognostic biomarkers and~~ ~~predictors of immunotherapy response. Higher ICI scores were associated with lower~~ ~~immune and matrix scores, while lower ICI scores correlated with greater immune~~ ~~infiltration, suggesting the utility of our subtype classification in guiding~~ ~~immunotherapy strategies. T~~o further stratify patients, we constructed an immune checkpoint inhibition (ICI) score, serving as a prognostic biomarker and a predictor of immunotherapy response. Notably, higher ICI scores were associated with lower immune and stromal scores, whereas lower ICI scores correlated with increased immune infiltration, suggesting the utility of our subtype classification in guiding immunotherapy strategies. Subtype C1 exhibited high immune scores and responsiveness to immune checkpoint inhibitors, while C2, with favorable prognosis and high ICI scores, may represent an earlier stage of CRC. This immune-based subtyping approach differs from broader classifications like the Consensus Molecular Subtypes(CMS)byspecificallytargeting LNM-negative CRC toguideimmunotherapy decisions. To reduce heterogeneity, our analysis was limited to LNM-negative cases; however, immune profiles may differ in LNM-positive patients. Further studies are needed to assess the generalizability of these subtypes across CRC stages.

Activation of the MAPK pathway through *KRAS* influences cell proliferation and differentiation, potentially preventing tumorigenesis [22]. CRCs with BRAF mutations is associated with lymphocyte infiltration and immune response activation [23], with studies indicating a positive correlation between PD-L1 expression and BRAF mutations. High CD8+ tumor-infiltrating lymphocytes observed in BRAF-mutant CRC suggest that these patients may respond well to immunotherapy [24]. Although *KRAS* mutations are generally unrelated to LNM status or tumor characteristics [4, 8], our study demonstrates a shift in *KRAS* mutation rates across the two LNM-negative

subtypes, potentially offering survival benefits to CRC patients with BRAF mutations.

To identifydiagnosticbiomarkersforCRCsubtypes,We~~ourstudy~~found that PPFIA4 was significantly upregulated in subtype C1 compared to C2~~expression is significantly~~ ~~upregulated in C1 compared to C2~~. Research on PPFIA4 in CRC is still in its early stages, and it may be associated with aberrant metabolic processes, positioning it as a target gene in cancer glycolysis [25]. Previous studies have confirmed that *PPFIA4* serves as a crucial molecular mechanism mediating intestinal carcinogenesis by influencing immune cell expression [26]. ~~Our findings reveal that CRC patients with~~ ~~elevated~~ *~~PPFIA4~~* ~~expression exhibit poorer overall survival. This study specifically~~ ~~evaluated the role of~~ *~~PPFIA4~~* ~~in the proliferation, migration, and invasion of colon~~ ~~cancer cells, establishing~~ *~~PPFIA4~~* ~~as a promising biomarker for subtype diagnosis in~~ ~~colon cancer.~~ In our study, elevated *PPFIA4* expression was associated with poorer overall survival, while its knockdown markedly inhibited CRC cell proliferation and migration, indicating a potential role in tumor progression. Beyond its biological function, *PPFIA4* holds promise as a clinically actionable biomarker, potentially serving both diagnostic and therapeutic purposes. Its subtype-specific expression profile could help distinguish more aggressive immune subtypes in early-stage CRC, and its inhibition may offer a new target for anti-tumor intervention. However, ~~our data~~ ~~are solely based on in vitro experiments, thus further in vivo studies are required to~~ ~~validate the efficacy of~~ *~~PPFIA4~~* ~~silencing in inhibiting cancer and enhancing survival~~ ~~rates, which can be explored through detailed investigations in human tissues and/or~~ ~~animal models.a~~s our findings are based solely on in vitro data, further validation through in vivo studies using patient tissues or animal models is necessary to confirm

its clinical utility.

**Formatted:** Font: (Default) Times New Roman, 12 pt

**Formatted:** Font: Italic

**Formatted:** Font: Italic

In conclusion, we conducted an in-depth analysis of the immune signature of LNM-negative CRC, especially regarding the relationship between immune subtypes and immunotherapy response. These findings enhance our understanding of CRC progression and may inform personalized strategies for cancer immunotherapy.

**Competing interests**

The author declares that he has no competing interests.

**~~Acknowledgements~~**

~~This work was partlysupportedbythe National NaturalScience Foundation of China~~ ~~(61775139 to Wenliang Yuan), and the Jiaxing Municipal Science and Technology~~ ~~Project (2023AY11057 to Wenliang Yuan and 2024AY10048 to Li Liu). There was no~~ ~~additional external funding received for this study. The funders had no role in study~~ ~~design, data collection and analysis, decision to publish, or preparation of the~~

~~manuscript.~~

**Ethics approval and consent to participate** Not applicable.

**Patient consent for publication** Not applicable.

**References**

1.	Bray F, Laversanne M, Sung H, Ferlay J, Siegel RL, Soerjomataram I, et al. Global cancer statistics 2022: GLOBOCAN estimates of incidence and mortality worldwide for 36 cancers in 185 countries. CA: a cancer journal for clinicians. 2024;74(3):229-63.

2.	Nancy K. The management of resectable and unresectable liver metastases from colorectal cancer. Current Opinion in Oncology. 2010;22(4):364-73.

3.	Lupinacci RM, Andraus W, Haddad LBDP, D ′	Albuquerque LAC, Herman P. Simultaneous laparoscopic resection of primary colorectal cancer and associated liver metastases: a systematic review. Techniques in Coloproctology. 2014;18(2):129-35.

4.	Yuan W, Li X, LiuL, Wei C, Sun D, Peng S, et al.Comprehensive analysis of lncRNA-associated ceRNA network in colorectal cancer. Biochem Biophys Res Commun 2019;508(2):374-9.

5.	Li R, Liu X, Huang X, Zhang D, Chen Z, Zhang J, et al. Single-cell transcriptomic analysis deciphers heterogenous cancer stem-like cells in colorectal cancer and their organ-specific metastasis. Gut. 2024;73(3):470-84.

6.	Dougan M, Dranoff G. Immune Therapy for Cancer. Annual Review of Immunology. 2009;27(27):83-117.

7.	Al Zein M, Boukhdoud M, Shammaa H, Mouslem H, El Ayoubi LM, Iratni R, et al. Immunotherapy and immunoevasion of colorectal cancer. Drug Discovery Today 2023;28(9):103669.

8.	Markman JL, Shiao SL. Impact of the immune system and immunotherapy in colorectal cancer. Journal of gastrointestinal oncology. 2015;6(2):208-23.

9.	Yuan W, Cai W, Huang X, Peng S. Prognostic value of immune scores in the microenvironment of colorectal cancer. Oncology Letters. 2020;20(5):1-.

10. Zhu X, Liang R, Lan T, Ding D, Huang S, Shao J, et al. Tumor-associated macrophage-specific CD155 contributes to M2-phenotype transition, immunosuppression, and tumor progression in colorectal cancer. Journal for immunotherapy of cancer. 2022;10(9). Epub 2022/09/15. doi: 10.1136/jitc-2021-004219. PubMed PMID: 36104099; PubMed Central PMCID: PMCPmc9476138.

11. Overman MJ, Mcdermott R, Leach JL, Lonardi S, André T. Nivolumab in patients with metastatic DNA mismatch repair-deficient or microsatellite instability-high colorectal cancer (CheckMate 142): an open-label, multicentre, phase 2 study. Lancet Oncology. 2017;18(9):1182-91.

12. Diaz LA, Jr., Shiu KK, Kim TW, Jensen BV, Jensen LH, Punt C, et al. Pembrolizumab versus chemotherapy for microsatellite instability-high or mismatch repair-deficient metastatic colorectal cancer (KEYNOTE-177): final analysis of a randomised, open-label, phase 3 study. The Lancet Oncology. 2022;23(5):659-70. Epub 2022/04/16. doi: 10.1016/s1470-2045(22)00197-8. PubMed PMID: 35427471; PubMed Central PMCID: PMCPmc9533375.

13. Thorsson V, Gibbs DL, Brown SD, Wolf D, Bortone DS, Yang T-HO, et al. The immune landscape of cancer. Immunity. 2018;48(4):812-30. e14.

14. Kursa MB, Rudnicki WR. Feature Selection with Boruta Package. Journal of Statistical Software. 2010;36(11):1-13.

15. Hnzelmann S, Castelo R, Guinney J. GSVA: gene set variation analysis for microarray and RNA-Seq data. BMC Bioinf 2013;14(1):7-.

16. Subramanian A, Tamayo P, Mootha VK, Mukherjee S, Ebert BL, Gillette MA, et al. Gene set enrichment analysis: a knowledge-based approach for interpreting genome-wide expression profiles. Proceedings of the National Academy of Sciences. 2005;102(43):15545-50.

17. Becht E, Giraldo NA, Lacroix L, Buttard… B. Estimating the population abundance of tissue-infiltrating immune and stromal cell populations using gene expression. Genome Biology. 2016;17(1):218.

18. Robinson MD, McCarthy DJ, Smyth GK. edgeR: a Bioconductor package for differential expression analysis of digital gene expression data. Bioinformatics. 2010;26(1):139-40.

19. Yoshihara K, Shahmoradgoli M, Martínez E, Vegesna R, Kim H, Torresgarcia W, et al. Inferring tumour purity and stromal and immune cell admixture from expression data. Nature Communications. 2013;4:2612.

20. Yu B, Kang J, Lei H, Li Z, Yang H, Zhang M. Immunotherapy for colorectal cancer. Front Immunol. 2024;15:1433315. Epub 2024/09/06. doi: 10.3389/fimmu.2024.1433315. PubMed PMID: 39238638; PubMed Central PMCID: PMCPmc11375682.

21. Wang X, Li M. Correlate tumor mutation burden with immune signatures in human cancers. BMC Immunology. 2019;20(1):4.

22. Sorokin AV, Kanikarla Marie P, Bitner L, Syed M, Woods M, Manyam G, et al. Targeting RAS Mutant Colorectal Cancer with Dual Inhibition of MEK and CDK4/6. Cancer Res. 2022;82(18):3335-44. Epub 2022/08/02. doi: 10.1158/0008-5472.can-22-0198. PubMed PMID: 35913398; PubMed Central PMCID: PMCPmc9478530.

23. Guinney J, Dienstmann R, Wang X, Reyniès A, Tejpar S. The consensus molecular subtypes of colorectal cancer. Nat Med 2015;21(11):1350-6.

24. Du W, Frankel TL, Green M, Zou W. IFNγ signaling integrity in colorectal cancer immunity and immunotherapy. Cellular & molecular immunology. 2022;19(1):23-32. Epub 2021/08/14. doi: 10.1038/s41423-021-00735-3. PubMed PMID: 34385592; PubMed Central PMCID: PMCPmc8752802. 25. Pate KT, Stringari C, Sprowl‐ Tanio S, Wang K, TeSlaa T, Hoverter NP, et al. Wnt signaling directs a metabolic program of glycolysisandangiogenesis in colon cancer. The EMBO journal.2014;33(13):1454-73.

26. Zheng FX, Yang CR, Sun FY, Zhang YZ, Wang YL, Li XZ, et al. Enterotoxin‐ related genes PPFIA4 and SCN3B promote colorectal cancer development and progression. J Biochem Mol Toxicol 2024;38(6):e23746.

**Supporting information**	**Formatted:** Heading 1

**S1 Table. The 109 immune associated genes used for classification. S2 Table. The result of differential expression analysis.**

**S3 Table. Functional enrichment analyses of subclass specific genes. S4 Table. Pathway enrichment analysis of two CRC subclasses.**

**S5 Table. P values for gene set mRNA enrichment analysis. S6 Table. The abundances of 13 immune-related cells.**

**S7 Table. Expression profiles of 19 potential targeted immune checkpoint genes in two subtypes.**

**S8 Table. TIDE value of immune checkpoint suppression therapy for each sample. S9 Table. Genetic mutation analysis of GSE39582 dataset.**

**S10 Table. ICI scores for each sample in TCGA and GSE39582.**

Response to Reviewers

**Journal Requirements:**

1.Please ensure that your manuscript meets PLOS ONE's style requirements, including those for file naming. The PLOS ONE style templates can be found at https://journals.plos.org/plosone/s/file?id=wjVg/PLOSOne_formatting_sample_main _body.pdf and

https://journals.plos.org/plosone/s/file?id=ba62/PLOSOne_formatting_sample_title_a uthors_affiliations.pdf

Response: Done.

2. Please note that funding information should not appear in any section or other areas of your manuscript. We will only publish funding information present in the Funding Statement section of the online submission form. Please remove any funding-related text from the manuscript.

Response: Done.

3. We note that the grant information you provided in the ‘Funding Information’ and ‘Financial Disclosure’ sections do not match.

When you resubmit, please ensure that you provide the correct grant numbers for the awards you received for your study in the ‘Funding Information’ section.

Response: Thank you for pointing out the discrepancy. We have reviewed and corrected the grant information to ensure consistency between the ‘Funding Information’ and ‘Financial Disclosure’ sections. The correct grant number(s) and funding agency details will be clearly provided in the revised submission.

4. When completing the data availability statement of the submission form, you indicated that you will make your data available on acceptance. We strongly recommend all authors decide on a data sharing plan before acceptance, as the process can be lengthy and hold up publication timelines. Please note that, though access restrictions are acceptable now, your entire data will need to be made freely accessible if your manuscript is accepted for publication. This policy applies to all data except where public deposition would breach compliance with the protocol approved by your research ethics board. If you are unable to adhere to our open data policy, please kindly revise your statement to explain your reasoning and we will seek the editor's input on an exemption. Please be assured that, once you have provided your new statement, the assessment of your exemption will not hold up the peer review process.

Response: Thank you for your reminder. We fully understand and respect PLOS ONE’s open data policy. We have prepared all underlying data and are currently organizing it for public deposition. Upon acceptance, we plan to make the complete

dataset freely accessible through an established public repository such as [e.g., Figshare, Dryad, GEO, or Zenodo]. We are committed to ensuring that all data necessary to replicate and validate our findings will be openly available prior to publication.

Should you require any further clarification or recommendations regarding the most suitable repository for our data type, we would be happy to comply.

5. Please include captions for your Supporting Information files at the end of your manuscript, and update any in-text citations to match accordingly. Please see our Supporting Information guidelines for more

information: [http://journals.plos.org/plosone/s/supporting-information.](http://track.editorialmanager.com/CL0/http:%2F%2Fjournals.plos.org%2Fplosone%2Fs%2Fsupporting-information/1/010f01969e3ebbe0-78bad238-6292-42e5-b879-fe5acd077e19-000000/2uSw4M0bjcIlyTcHd1F4RzE3tEhMKSAew0nkxQ3ljyA=209)

Response: Done.

6. PLOS ONE now requires that authors provide the original uncropped and unadjusted images underlying all blot or gel results reported in a submission’s figures or Supporting Information files. This policy and the journal’s other requirements for blot/gel reporting and figure preparation are described in detail

at <https://journals.plos.org/plosone/s/figures#loc-blot-and-gel-reporting-requirements> and [https://journals.plos.org/plosone/s/figures#loc-preparing-figures-from-image-files.](https://journals.plos.org/plosone/s/figures#loc-preparing-figures-from-image-files) When you submit your revised manuscript, please ensure that your

figures adhere fully to these guidelines and provide the original underlying images for all blot or gel data reported in your submission. See the following link for instructions on providing the original image data: [https://journals.plos.org/plosone/s/figures#loc-original-images-for-blots-and-gels.](https://journals.plos.org/plosone/s/figures#loc-original-images-for-blots-and-gels)

In your cover letter, please note whether your blot/gel image data are in Supporting Information or posted at a public data repository, provide the repository URL if relevant, and provide specific details as to which raw blot/gel images, if any, are not available. Email us at plosone@plos.org if you have any questions.

Response: We have prepared the original, uncropped, and unadjusted images underlying all blot and gel results reported in the manuscript. These files have been compiled into a PDF document and are provided as Supporting Information in accordance with the journal’s guidelines. Please let us know if further formatting or additional information is required.

**Reviewers' comments:**

1. Is the manuscript technically sound, and do the data support the conclusions?

The manuscript must describe a technically sound piece of scientific research with data that supports the conclusions. Experiments must have been conducted rigorously, with appropriate controls, replication, and sample sizes. The conclusions must be drawn appropriately based on the data presented.

Reviewer #1: Yes

Reviewer #2: Yes

2. Has the statistical analysis been performed appropriately and rigorously?

Reviewer #1: I Don't Know

Reviewer #2: Yes

3. Have the authors made all data underlying the findings in their manuscript fully available?

The [PLOS Data policy](http://www.plosone.org/static/policies.action#sharing) requires authors to make all data underlying the findings described in their manuscript fully available without restriction, with rare exception (please refer to the Data Availability Statement in the manuscript PDF file). The data should be provided as part of the manuscript or its supporting information, or deposited to a public repository. For example, in addition to summary statistics, the data points behind means, medians and variance measures should be available. If there are restrictions on publicly sharing data—e.g. participant privacy or use of data from a third party—those must be specified.

Reviewer #1: Yes

Reviewer #2: Yes

4. Is the manuscript presented in an intelligible fashion and written in standard English?

PLOS ONE does not copyedit accepted manuscripts, so the language in submitted articles must be clear, correct, and unambiguous. Any typographical or grammatical errors should be corrected at revision, so please note any specific errors here.

Reviewer #1: Yes

Reviewer #2: Yes

5. Review Comments to the Author

Please use the space provided to explain your answers to the questions above. You may also include additional comments for the author, including concerns about dual publication, research ethics, or publication ethics. (Please upload your review as an attachment if it exceeds 20,000 characters)

Reviewer #1: This manuscript describes an interesting and relevant study on immune subtyping of lymph node metastasis-negative colorectal cancer, which holds great promise for providing biomarkers to predict prognosis and response to immunotherapy. The experimental design seems appropriate, and the conclusions drawn are generally supported by the data.However, I recommend statistical analysis experts who can better comment on this aspect, given that I am not a statistics professional and cannot fully evaluate the rigor of this component.

Recommendations:

• The methodology should discuss quite well the choice of specific machine learning algorithms.

Response: We appreciate the reviewer’s insightful comment. In the revised Materials and Methods section, we have clarified the rationale for selecting each machine learning algorithm within the Materials and Methods section. Specifically, we used NMF for its effectiveness in identifying biologically meaningful, non-overlapping expression patterns for cancer subtyping. PCA was then employed to construct the ICI score by extracting principal components that summarize key feature dimensions into interpretable scores. These revisions now better justify our methodological choices.

• Compare the results with those of existing classifications of colorectal cancer

Response: Thank you for the helpful suggestion. A brief comparison with existing classification systems, such as CMS, has been added at the end of the first paragraph of the Discussion section. Our findings indicate that the proposed immune-based subtyping offers more precise stratification, particularly for LNM-negative CRC patients, and serves as a complementary approach to the broader transcriptomic-based classifications.

• Strengthen the discussion of clinical applications, the sense of the clinical contributions of PPFIA4 and ICI scores should be amplified.

Response: We appreciate the reviewer’s suggestion. In the revised Discussion section, we have expanded the discussion on the clinical implications of PPFIA4 and the ICI score. Specifically, PPFIA4 is highlighted as a potential diagnostic and therapeutic target, given its subtype-specific expression profile and involvement in CRC

progression. Moreover, the ICI score is emphasized as a practical immune-related metric, capable of identifying LNM-negative patients who may derive benefit from immunotherapy. This metric may complement current biomarkers and guide personalized treatment decisions. These revisions underscore the translational potential of our findings.

• Improve the quality of the figures, as they appear blurry.

Response: We thank the reviewer for pointing out the issue regarding figure clarity. In response, we have replaced the affected figures with high-resolution versions to ensure clarity and compliance with publication standards. We believe these improvements substantially enhance the overall visual presentation of our data.

Reviewer #2: Dear authors,

Thank you for submitting this interesting and timely research. Please find below my comments aimed at improving clarity and strengthening the manuscript:

Clarification of Subtype Characteristics:

The subtypes C1 and C2 are defined, but their prognostic and immunological implications remain somewhat unclear. Both are described as having "high immune infiltration" or "favorable prognosis," which can be confusing. Please consider clearly and consistently presenting the defining features—both prognostic and immunological—of each subtype early in the manuscript and maintain this consistency throughout.

Contextualization with Existing Classifications:

It would be helpful to expand on how the C1/C2 subtypes align with or differ from existing colorectal cancer immune classifications, such as the CMS (Consensus Molecular Subtypes). Additionally, consider discussing the broader relevance to immunotherapy beyond PD-1/PD-L1, and how the ICI scores proposed in your study might guide treatment decisions in clinical practice.

Limitations Section:

Please include a dedicated limitations section and elaborate on the following points: The lack of in vivo validation for PPFIA4.

Response: We thank the reviewer for highlighting this important limitation. As our current study primarily employed in vitro assays, we now explicitly acknowledge in the Discussion that in vivo validation of PPFIA4’s functional role and therapeutic relevance remains essential. We have also outlined the need for future studies using animal models or patient-derived samples to confirm our findings. This acknowledgment clarifies the scope of the current study and sets a clear direction for future research.

The potential for dataset bias (GEO vs. TCGA).

Response: We thank the reviewer for pointing out the important issue of potential dataset bias between TCGA and GEO. To minimize such bias, we performed the following measures:

1)Independent Subtype Identification and Validation: The immune subtypes (C1 and C2) were separately identified in the TCGA dataset and independently validated in two GEO datasets (GSE39582 and GSE103479). The consistent results across datasets (confirmed by NMF clustering, t-SNE analysis, and survival differences) demonstrate the robustness and reproducibility of the subtype classification.

2)Standard Preprocessing: All datasets underwent standardized preprocessing procedures, including normalization and log2 transformation. Low-variance genes were removed prior to clustering to reduce noise.

3)Cross-dataset Consistency: In both TCGAand GEO cohorts, the subtypes showed similar immune characteristics, prognostic trends, and immune checkpoint profiles, which reduces the likelihood of dataset-specific artifacts.

4)Additional Clarification: In the revised manuscript (Results section), we have added a brief explanation on how dataset bias was addressed, and emphasized the use of independent validation cohorts to strengthen our conclusions.

These steps enhance confidence in the reliability and transferability of our findings.

The generalizability of your findings across diverse CRC patient populations. Response: We thank the reviewer for raising this important point.. Our study specifically focused on LNM-negative CRC patients to minimize confounding factors associated with advanced disease stages. We acknowledge that the immune microenvironment may differ significantly in LNM-positive patients, potentially affecting the applicability of our identified immune subtypes. We have addressed this limitation in the revised manuscript and emphasized the need for future studies to validate our findings in broader CRC populations, including those with lymph node involvement.

Language and Formatting:

The manuscript would benefit from a careful grammar and spell check, as well as the correction of typographical errors throughout.

Response: We thank the reviewer for noting the need for language and formatting improvements. In response, we have thoroughly reviewed the manuscript to correct

grammatical errors, spelling mistakes, and typographical issues.Additionally, we have ensured consistencyin formatting throughout the document. We believe these revisions have enhanced the clarity and readability of the manuscript.

6. PLOS authors have the option to publish the peer review history of their article [(what does this mean?)](https://journals.plos.org/plosone/s/editorial-and-peer-review-process#loc-peer-review-history). If published, this will include your full peer review and any attached files.

If you choose “no”, your identity will remain anonymous but your review may still be made public.

**Do you want your identity to be public for this peer review?** For information about this choice, including consent withdrawal, please see our [Privacy Policy.](https://www.plos.org/privacy-policy)

Reviewer #1: No

Reviewer #2: No
