# Supplementary material for: Immune subtyping of lymph node metastasis-negative colorectal cancer reveals biomarkers for prognosis and immunotherapy response
Source: PLoS One. 2025 Sep 24;20(9):e0332915. doi: 10.1371/journal.pone.0332915 (PMC12459767; doi:10.1371/journal.pone.0332915)
Supplement: Attachment — Submitted filename: Response_to_Reviewers_auresp_2.docx [file pone.0332915.s016.docx]

**Journal Requirements:**

Please review your reference list to ensure that it is complete and correct. If you have cited papers that have been retracted, please include the rationale for doing so in the manuscript text, or remove these references and replace them with relevant current references. Any changes to the reference list should be mentioned in the rebuttal letter that accompanies your revised manuscript. If you need to cite a retracted article, indicate the article’s retracted status in the References list and also include a citation and full reference for the retraction notice.

Response: We appreciate the editor’s reminder regarding the integrity and completeness of the reference list.

We have carefully reviewed all references cited in our manuscript. As of the date of this revision, none of the cited articles have been retracted, nor are there any associated expressions of concern or correction notices according to searches in PubMed, the Retraction Watch Database, CrossRef, and the official websites of the respective journals.

Therefore, no retracted articles remain in the reference list, and no changes are needed in this regard. A full check of reference validity has also been documented as part of our revision process.

Should any changes to the reference list be made in future revisions, we will ensure they are appropriately reflected in both the manuscript and the rebuttal letter.

**Reviewers' comments:**
1. If the authors have adequately addressed your comments raised in a previous round of review and you feel that this manuscript is now acceptable for publication, you may indicate that here to bypass the “Comments to the Author” section, enter your conflict of interest statement in the “Confidential to Editor” section, and submit your "Accept" recommendation.

Reviewer #2: (No Response)

Reviewer #3: (No Response)

Reviewer #4: (No Response)

2. Is the manuscript technically sound, and do the data support the conclusions?

The manuscript must describe a technically sound piece of scientific research with data that supports the conclusions. Experiments must have been conducted rigorously, with appropriate controls, replication, and sample sizes. The conclusions must be drawn appropriately based on the data presented.

Reviewer #2: Yes

Reviewer #3: Yes

Reviewer #4: Partly

3. Has the statistical analysis been performed appropriately and rigorously?

Reviewer #2: Yes

Reviewer #3: Yes

Reviewer #4: I Don't Know

4. Have the authors made all data underlying the findings in their manuscript fully available?

The [PLOS Data policy](http://track.editorialmanager.com/CL0/http:%2F%2Fwww.plosone.org%2Fstatic%2Fpolicies.action%23sharing/1/010f01982ab62aea-0699af18-7413-4f20-a1a2-bfc67a2cddef-000000/FaHE0FSb5G4p2u403FOg9GBvZ9cv5NRvYylyuz2980k=220) requires authors to make all data underlying the findings described in their manuscript fully available without restriction, with rare exception (please refer to the Data Availability Statement in the manuscript PDF file). The data should be provided as part of the manuscript or its supporting information, or deposited to a public repository. For example, in addition to summary statistics, the data points behind means, medians and variance measures should be available. If there are restrictions on publicly sharing data—e.g. participant privacy or use of data from a third party—those must be specified.

Reviewer #2: Yes

Reviewer #3: Yes

Reviewer #4: Yes

5. Is the manuscript presented in an intelligible fashion and written in standard English?

PLOS ONE does not copyedit accepted manuscripts, so the language in submitted articles must be clear, correct, and unambiguous. Any typographical or grammatical errors should be corrected at revision, so please note any specific errors here.

Reviewer #2: Yes

Reviewer #3: Yes

Reviewer #4: Yes

6. Review Comments to the Author

Please use the space provided to explain your answers to the questions above. You may also include additional comments for the author, including concerns about dual publication, research ethics, or publication ethics. (Please upload your review as an attachment if it exceeds 20,000 characters)

Reviewer #2: (No Response)

Reviewer #3: In this study, the authors investigated the LNM characteristics that may serve as prognistic markers for CRC and immunotherapy efficacy. They found that subtype C1 showed high immune scores and responsiveness to ICB, while C2 was correlated with more favourable diagnoses and immune cell infiltration. They also found that PPFIA4 KD in C1 suppressed CRC cell proliferation and migration

I'd like to congratulate the authors on their manuscript and very interesting findings. I found it to be well written and the results were clearly communicated. I can see that the authors effectively incoorpated previous feedback. I have added a couple additional comments in the form of track changes in the attached document. With these small changes, I believe this manuscript is ready for publication

Reviewer #4:

1- It is recommended to separate Figure 1 panels a–c from panels d–f to enhance clarity. For example, adding subtitles such as “TCGA” above panels a–c and “GSE” above panels d–f would facilitate comprehension.

Response: Thank you for your helpful suggestion regarding Figure 1. While we chose not to separate panels a–c and d–f into two distinct figures in order to maintain visual continuity and comparative consistency, we fully agree that indicating the dataset source would enhance clarity. Accordingly, we have added subtitles “TCGA” and “GSE39582” directly above the relevant panel groups in Figure 1 to clearly indicate the corresponding datasets. This adjustment improves figure clarity while preserving comparative consistency.

2- It is recommended to have the analysis workflow reviewed by a biostatistician to ensure robust validation of the statistical and computational methods employed.

Response: Thank you for your valuable comment regarding the validation of statistical and computational methods. To address this point, we have consulted with a professional biostatistician to review our analytical workflow and confirm the appropriateness of the applied statistical techniques. Accordingly, we have revised the “Statistical analyses” section of the manuscript to explicitly state that the analysis pipeline was reviewed in consultation with a biostatistical expert. This addition enhances methodological transparency.

3- In the Introduction, include additional background information on LNM-negative CRC from the literature, as this is the focus of the study. Providing more context would benefit readers prior to the authors’ subtyping analysis.

Response: We thank the reviewer for this thoughtful suggestion. To provide additional context for our subtyping analysis, we have revised the Introduction to include relevant background information on LNM-negative colorectal cancer (CRC). Specifically, we now discuss the clinical relevance, heterogeneity, and limited immunological characterization of this CRC subgroup in the Introduction, which highlights the need for comprehensive immune subtyping.

4- Specify the meaning of the abbreviation “LN” somewhere in the manuscript. At the beginning of the paper, lymph node metastasis-negative colorectal cancer is referred to as “LNM CRC,” but at a certain point, it is abbreviated simply as “LN.” In this regard, please ensure that all acronyms are defined upon their first use, as some are currently missing explanations. For example, when PPFIA4 is mentioned, the name of the corresponding protein should be presented alongside the gene nomenclature, since it only appears in the following paragraph.

Response: We appreciate the reviewer’s careful attention to acronym consistency. In response, we have revised the manuscript to uniformly refer to “lymph node metastasis-negative colorectal cancer” as LNM-negative CRC throughout the text. All previous instances of “LN-negative CRC” or similar variants have been corrected accordingly to maintain terminological clarity and consistency. We have also updated the initial mention of “PPFIA4” to include the corresponding protein name “liprin-α4.” Furthermore, we performed a thorough review to ensure that all abbreviations are clearly defined at their first appearance in the manuscript.

5- In the paragraph titled “Correlation of the LN-negative CRC Subtypes With Immune-Associated Signatures,” please add a brief description or definition of the scores mentioned. A detailed explanation is not necessary, but including a few clarifying words about what the “cytolytic score”, “stromal score”, etc. represent would enhance the reader’s understanding and improve the overall flow of the text.

Response: We appreciate the reviewer’s suggestion to clarify the scoring terms. In the revised Results section titled “Correlation of the LNM-negative CRC Subtypes With Immune-Associated Signatures,” we have added concise explanatory notes for these scores to enhance clarity without disrupting the flow.

6- It is not clear to me why PPFIA4 is introduced abruptly in the manuscript. I suggest providing some background or rationale explaining why the authors chose to investigate this particular gene. In the Discussion, the authors state “To identify diagnostic biomarkers for CRC subtypes, We found that PPFIA4 was significantly upregulated in subtype C1 compared to C2.” However, I am unable to locate any corresponding figure or graph supporting this statement. I kindly ask the authors to clarify the origin of this result or to include additional information in the manuscript to justify the focus on PPFIA4.

Response: We thank the reviewer for raising this important point regarding the rationale for focusing on PPFIA4 and the clarity of its presentation. In response, we have revised the Results section to explicitly state that PPFIA4 was identified through differential gene expression analysis as one of the top upregulated genes in subtype C1 (now referenced in S2 Table). We also added a brief sentence to explain that this finding prompted further investigation into its potential biological function. In the Discussion section, we expanded the rationale by introducing background information on PPFIA4 and its known role in tumor metabolism and immune regulation, citing relevant literature ([21], [22]). This revision better contextualizes our decision to study this gene and supports its relevance as a potential subtype-specific biomarker and therapeutic target. These revisions clarify our rationale for focusing on PPFIA4 and substantiate its relevance as a candidate biomarker.

7- In the Materials and Methods section, please provide a more detailed description of the wound healing assay methodology. The phrase “confluent cells were scratched” is insufficient, as the type of scratch performed can greatly influence the results; for example, whether the scratch was made manually with a pipette tip (which may produce irregular edges and complicate statistical analysis), with an IncuCyte WoundMaker, or using a silicone spacer. Additionally, please clarify whether mitomycin was added to the wound assay to inhibit cell proliferation. This detail is important to demonstrate that the observed reduction in the wound gap after 48 hours (when cells are silenced) is not solely due to proliferation effects, especially since the MTT assay indicates that HCT-116 cells exhibit reduced proliferation after two days.

Response: Thank you for the constructive suggestion. Detailed methodological information—including use of a pipette tip, mitomycin C treatment, and image quantification via ImageJ—has been added to the Materials and Methods section. The Results text remains unchanged to preserve clarity and continuity.

8- In the Materials and Methods section , please specify the number of replicates that have been used for the in vitro experiments.

Response: We appreciate the reviewer’s comment regarding the clarification of replicate numbers. In the revised Materials and Methods section, we have now specified the number of biological and technical replicates used for each in vitro experiment, including the MTT assay, wound healing assay, qPCR, and Western blot. All experiments were performed in at least three independent biological replicates to ensure statistical robustness and reproducibility.

7. PLOS authors have the option to publish the peer review history of their article ([what does this mean?](https://track.editorialmanager.com/CL0/https:%2F%2Fjournals.plos.org%2Fplosone%2Fs%2Feditorial-and-peer-review-process%23loc-peer-review-history/1/010f01982ab62aea-0699af18-7413-4f20-a1a2-bfc67a2cddef-000000/7IuqBxFOVCK9I40seHLfQv2-GQcN2jEV6mqBhxpGG2s=220)). If published, this will include your full peer review and any attached files.

If you choose “no”, your identity will remain anonymous but your review may still be made public.

**Do you want your identity to be public for this peer review?** For information about this choice, including consent withdrawal, please see our [Privacy Policy](https://track.editorialmanager.com/CL0/https:%2F%2Fwww.plos.org%2Fprivacy-policy/1/010f01982ab62aea-0699af18-7413-4f20-a1a2-bfc67a2cddef-000000/2TZ0zTcFfFkqoi1h19AgOa6f9Zc08dp-NQQEBG9VI5Q=220).

Reviewer #2: No

Reviewer #3: No

Reviewer #4: No

**Additional Revisions Based on Editorial Suggestions:**
In addition to addressing the reviewers’ comments, we have also carefully reviewed and implemented suggestions provided in the editorial checklist and manuscript preparation guidelines. Specifically, we:

- Defined the abbreviation “TNM” at its first occurrence in the Introduction;
- Removed the redundant definition of “ICI” in the Discussion section, as it was already defined in the Introduction.

These changes have been incorporated into the revised manuscript to improve clarity and consistency.
